# Socioeconomic Inequalities in Body Mass Index across Adulthood: Coordinated Analyses of Individual Participant Data from Three British Birth Cohort Studies Initiated in 1946, 1958 and 1970

**DOI:** 10.1371/journal.pmed.1002214

**Published:** 2017-01-10

**Authors:** David Bann, William Johnson, Leah Li, Diana Kuh, Rebecca Hardy

**Affiliations:** 1 Centre for Longitudinal Studies, UCL Institute of Education, London, United Kingdom; 2 School of Sport, Exercise and Health Sciences, Loughborough University, Loughborough, United Kingdom; 3 Population, Policy and Practice, UCL Institute of Child Health, London, United Kingdom; 4 MRC Unit for Lifelong Health and Ageing at UCL, London, United Kingdom; Stanford University, UNITED STATES

## Abstract

**Background:**

High body mass index (BMI) is an important contributor to the global burden of ill-health and health inequality. Lower socioeconomic position (SEP) in both childhood and adulthood is associated with higher adult BMI, but how these associations have changed across time is poorly understood. We used longitudinal data to examine how childhood and adult SEP relates to BMI across adulthood in three national British birth cohorts.

**Methods and Findings:**

The sample comprised up to 22,810 participants with 77,115 BMI observations in the 1946 MRC National Survey of Health and Development (ages 20 to 60–64), the 1958 National Child Development Study (ages 23 to 50), and the 1970 British Cohort Study (ages 26 to 42). Harmonized social class-based SEP data (Registrar General’s Social Class) was ascertained in childhood (father’s class at 10/11 y) and adulthood (42/43 years), and BMI repeatedly across adulthood, spanning 1966 to 2012. Associations between SEP and BMI were examined using linear regression and multilevel models.

Lower childhood SEP was associated with higher adult BMI in both genders, and differences were typically larger at older ages and similar in magnitude in each cohort. The strength of association between adult SEP and BMI did not vary with age in any consistent pattern in these cohorts, but were more evident in women than men, and inequalities were larger among women in the 1970 cohort compared with earlier-born cohorts. For example, mean differences in BMI at 42/43 y amongst women in the lowest compared with highest social class were 2.0 kg/m^2^ (95% CI: −0.1, 4.0) in the 1946 NSHD, 2.3 kg/m^2^ (1.1, 3.4) in the 1958 NCDS, and 3.9 kg/m^2^ (2.3, 5.4) the in the 1970 BCS; mean (SD) BMI in the highest and lowest social classes were as follows: 24.9 (0.8) versus 26.8 (0.7) in the 1946 NSHD, 24.2 (0.4) versus 26.5 (0.4) in the 1958 NCDS, and 24.2 (0.3) versus 28.1 (0.8) in the 1970 BCS. Findings did not differ whether using overweight or obesity as an outcome.

Limitations of this work include the use of social class as the sole indicator of SEP—while it was available in each cohort in both childhood and adulthood, trends in BMI inequalities may differ according to other dimensions of SEP such as education or income. Although harmonized data were used to aid inferences about birth cohort differences in BMI inequality, differences in other factors may have also contributed to findings—for example, differences in missing data.

**Conclusions:**

Given these persisting inequalities and their public health implications, new and effective policies to reduce inequalities in adult BMI that tackle inequality with respect to both childhood and adult SEP are urgently required

## Abstract 2

In a harmonized analysis of socioeconomic and anthropometric data from three cohorts, David Bann and colleagues trace the relationship between socioeconomic status and BMI over time in the UK.

## Author Summary

**Why Was This Study Done?:**

- High body mass index (BMI) is thought to be harmful to human health—in most adults, a high BMI is due to having high amounts of fat mass in the body.
- Previous studies have found that those with fewer socioeconomic resources—both as children and as adults—are more likely, on average, to have a higher BMI as adults.
- Reducing these socioeconomic inequalities in BMI is an important health policy goal, yet there is limited existing data to help us understand comprehensively how these inequalities have changed across time.

**What Did the Researchers Do and Find?:**

- We used data from three national British birth cohort studies of those born in 1946, 1958, and 1970—these studies contain comparable data on social class in childhood and adulthood, and on BMI across adult life.
- We confirm that large inequalities in BMI exist, according to both childhood and adult SEP—these were stronger among women, but also found among men.
- Inequalities according to childhood SEP generally become progressively larger at older ages in all cohorts and in both genders; inequalities according to adult SEP were larger among more recently born generations of women.

**What Do These Findings Mean?:**

- The fact that BMI inequalities have persisted or increased across different generations, despite policies designed to reduce them, suggests that new policies are required.
- Results support the need to intervene earlier rather than later in adult life, since inequalities tend to become larger at older ages.
- Limitations include the use of only one aspect of socioeconomic circumstances (social class), and the fact that not all participants continue to provide data in longitudinal studies—this may have led us to underestimating the size of BMI inequalities.

## Introduction

High body mass index (BMI) is an important modifiable contributor to the global burden of ill-health and health expenditure,[1, 2] and its prevalence increased markedly between the 1980s and 2014.[3–5] National attempts to reduce population BMI levels have thus far largely been unsuccessful,[3–5] suggesting that it is likely to be an important threat to the health of future generations.[3] Indeed, the increasing prevalence of high BMI at younger ages suggests that later-born generations are at risk of spending longer periods of life either overweight or obese.[6] Systematic reviews have also shown that in high income countries, lower socioeconomic position (SEP) in childhood and adulthood are associated with higher adult BMI and increased obesity risk.[7–9] Due to the links between higher BMI and adverse health outcomes,[10, 11] inequalities in BMI are likely to be an important contributor to socioeconomic inequalities in health. Accordingly, reducing BMI inequalities is a stated goal of numerous health policymakers and organizations; [12] achieving this requires high-quality evidence on how such inequalities have changed across time, in response to changing policy and economic contexts.

Existing evidence on how inequalities in BMI have changed across generations is largely restricted to repeated analyses of cross-sectional data.[13–21] These studies have tended to report persisting inequalities in BMI that are stronger among women, yet are limited to investigating relatively recent changes (e.g., from 1993/1994 to 2002/2003,[13] or 1994 to 2008[14]). They also do not elucidate how inequalities in BMI change with age; understanding these patterns may help inform the development of interventions targeted at the most effective ages. Such differences may reflect age differences in susceptibility to weight gain, which in turn may differ by cohort depending on the period of life when exposed to more obesogenic environments. The use of repeated cross-sectional adult data also leads to an almost exclusive focus on the changing consequences of adult SEP in previous studies, since such studies do not have (by design) prospective measures of childhood SEP. However, childhood socioeconomic circumstances strongly determine those in adulthood, and childhood SEP has been repeatedly related to higher adult adiposity and other health outcomes independent of adult SEP.[22–27] Relations between childhood SEP and adult BMI are also less likely to be affected by reverse causation than those with adult SEP, since obesity may impair adult economic outcomes;[28–31] childhood SEP is therefore an important dimension of socioeconomic circumstances to consider when understanding how inequalities have changed across time. Many previous studies have not investigated inequality on both relative (e.g., % change) and absolute (difference in kg/m^2^) scales. Since changes in relative inequality can occur despite opposing or no changes in absolute inequality, particularly when dichotomized outcomes are used and the overall population outcome prevalence changes, analyzing both is likely to be important in order to better understand how inequalities have changed across time, and their population health impact.[32, 33] To our knowledge, the literature currently lacks a comprehensive coordinated analysis to investigate how inequalities in BMI across adulthood have changed over the course of the obesity epidemic, with respect to both childhood and adult SEP.

The objectives of this study were to examine trends in the socioeconomic distribution of BMI and overweight or obesity across adult life, using data from the British birth cohort studies initiated in 1946, 1958, and 1970. These data have previously been used to show that the obesity epidemic has hit more recently born generations at increasingly younger ages in adulthood;[6] as with evidence from repeated cross-sectional studies,[4] this paper found that obesity prevalence increased markedly from the 1980s onwards in the United Kingdom and remained persistently high up to 2012. We use data collected between 1966 and 2012, which covers this period. Consistent with a fundamental cause hypothesis for understanding health inequality,[34, 35] we expected that due to the increasing public dissemination of the health harms of obesity, those of higher SEP may have been increasingly able to use their greater social, financial, and educational resources to protect themselves against excessive weight gain across adulthood; principally by modifying their diet and/or physical activity levels. As such, we hypothesized that socioeconomic inequalities in BMI would be larger in cohorts born later in the 20th century, and that these differences would be evident for both childhood and adult SEP.

## Methods

### Study samples

Each study used in this manuscript has received relevant ethical approval and obtained parental/participant consent; this information is available from the study websites and/or cohort profiles.

Britain’s birth cohort studies with participants followed up through adulthood were used. These were designed to be nationally representative when initiated in 1946 (MRC National Survey of Health and Development[36, 37]—1946 NSHD), 1958 (National Child Development Study[38]—1958 NCDS), and 1970 (British Cohort Study[39]—1970 BCS). The history, design, and characteristics of these studies have been previously described in detail in papers[36–40] and books;[41, 42] studies have also examined the characteristics of those lost due to attrition.[43–46] To aid the comparability of associations between SEP and BMI across studies, analyses were restricted to singleton births in England, Scotland, and Wales from those born and included in cohorts in the relevant weeks in March/April 1946 (*N* = 5,362), 1958 (*N* = 16,383), and 1970 (*N* = 16,172). In the 1946 NSHD, participants were restricted to those from married mothers due to tracing difficulties in the minority of babies born to unmarried mothers.[40] The weeks of initiation were chosen on the basis of practical considerations at the time of study development. The analytic sample sizes were those with valid data for each SEP measure and at least one adult BMI measure; for childhood SEP, a total of 22,810 participants with 77,115 BMI observations; for adult SEP, a total of 17,898 participants with 36,702 BMI observations (sample sizes for all analyses, in each cohort, age, and gender are shown in Table 1 and Table 2, and in S1 Table and S2 Table). All participants in the 1946 NSHD were white, as were 98.7% of those in the 1958 NCDS, and 95.4% of 1970 BCS. All analyses using the 1946 NSHD were weighted to account for the stratified sampling design[37].

**Table 1:** Father’s social class (10/11 y) and BMI across adulthood in the 1946 NSHD, the 1958 NCDS, and the 1970 BCS British birth cohort studies

			kg/m^2^ BMI difference (95% CI)					
Cohort	*Gender*, *age*							
1946 NSHD	Men	*N*	I (ref)	II	III NM	III M	IV	V
	20	1,562	-	0.6 (0.0, 1.2)	0.3 (−0.3, 0.9)	0.9 (0.3, 1.4)	0.9 (0.3, 1.5)	1.4 (0.7, 2.2)
	26	1,552	-	0.7 (0.1, 1.3)	0.6 (−0.1, 1.3)	1.2 (0.6, 1.7)	1.3 (0.6, 2.0)	1.7 (0.9, 2.5)
	36	1,365	-	0.9 (0.1, 1.8)	0.7 (−0.2, 1.6)	1.6 (0.8, 2.4)	1.8 (0.9, 2.7)	1.5 (0.4, 2.6)
	43	1,338	- #	0.9 (0.1, 1.8)	0.8 (−0.1, 1.7)	1.7 (0.9, 2.4)	1.9 (1.0, 2.8)	1.3 (0.2, 2.3)
	53	1,206	- #	1.0 (0.0, 2.0)	0.7 (−0.3, 1.8)	1.9 (1.0, 2.9)	2.2 (1.1, 3.3)	1.5 (0.3, 2.8)
	60–64	898	- #	0.8 (−0.4, 2.0)	0.7 (−0.5, 1.8)	2.2 (1.2, 3.3)	2.3 (1.0, 3.5)	1.1 (−0.5, 2.8)
1958 NCDS	23	4,046	-	0.4 (−0.1, 0.8)	0.3 (−0.2, 0.8)	0.8 (0.4, 1.3)	0.8 (0.3, 1.3)	0.9 (0.4, 1.4)
	33	3,547	-	0.3 (−0.3, 1.0)	0.6 (0.0, 1.3)	0.9 (0.3, 1.5)	1.0 (0.4, 1.7)	0.9 (0.2, 1.6)
	42	3,637	-	0.7 (0.0, 1.3)	0.7 (0.0, 1.4)	1.0 (0.4, 1.6)	1.2 (0.5, 1.9)	1.4 (0.7, 2.2)
	44	3,046	-	0.7 (−0.1, 1.4)	0.8 (0.0, 1.7)	1.2 (0.5, 1.9)	1.6 (0.8, 2.4)	1.8 (0.9, 2.6)
	50	2,766	-	1.0 (0.1, 1.8)	1.1 (0.2, 2.1)	1.6 (0.8, 2.4)	2.2 (1.3, 3.1)	2.1 (1.2, 3.1)
1970 BCS	26	1,923	-	0.3 (−0.4, 1.0)	0.0 (−0.8, 0.7)	0.8 (0.1, 1.4)	1.1 (0.4, 1.9)	1.2 (0.3, 2.1)
	30	3,960	-	0.4 (−0.1, 1.0)	0.3 (−0.4, 0.9)	0.8 (0.3, 1.3)	0.6 (0.0, 1.2)	0.7 (0.0, 1.4)
	34	3,435	-	0.4 (−0.2, 1.0)	0.4 (−0.3, 1.1)	1.0 (0.4, 1.6)	1.1 (0.4, 1.8)	1.2 (0.4, 1.9)
	42	3,208	-	0.4 (−0.4, 1.1)	0.6 (−0.2, 1.5)	1.3 (0.6, 2.0)	1.3 (0.5, 2.1)	0.9 (0.0, 1.9)
1946 NSHD	Women	N	I (ref)	II	III NM	III M	IV	V
	20	1,426	-	0.3 (−0.4, 1.0)	−0.2 (−0.8, 0.5)	0.3 (−0.3, 0.9)	0.8 (0.1, 1.4)	0.9 (0.0, 1.9)
	26	1,543	-	0.5 (−0.2, 1.2)	0.3 (−0.4, 0.9)	0.9 (0.3, 1.5)	1.4 (0.7, 2.1)	1.7 (0.6, 2.7)
	36	1,353	- #	0.5 (−0.4, 1.4)	−0.1 (−0.9, 0.8)	1.3 (0.5, 2.2)	2.0 (1.0, 2.9)	1.7 (0.5, 2.9)
	43	1,315	-	0.5 (−0.6, 1.6)	0.2 (−0.8, 1.2)	1.4 (0.4, 2.4)	2.2 (1.1, 3.3)	1.7 (0.2, 3.2)
	53	1,235	-	0.8 (−0.5, 2.2)	0.5 (−0.9, 1.8)	1.7 (0.4, 2.9)	3.0 (1.6, 4.4)	1.6 (−0.4, 3.6)
	60–64	958	- #	1.1 (−0.3, 2.6)	0.2 (−1.2, 1.6)	1.6 (0.3, 2.8)	3.6 (2.1, 5.1)	3.1 (0.6, 5.7)
1958 NCDS	23	4,115	- #	0.6 (0.1, 1.2)	0.5 (0.0, 1.1)	1.4 (0.9, 1.9)	1.3 (0.8, 1.9)	1.2 (0.6, 1.8)
	33	3,524	- #	0.7 (−0.2, 1.5)	0.9 (0.0, 1.8)	1.9 (1.1, 2.7)	1.9 (1.0, 2.8)	1.3 (0.3, 2.3)
	42	3,668	- #	0.8 (−0.1, 1.6)	0.8 (−0.1, 1.7)	2.0 (1.2, 2.8)	2.3 (1.4, 3.1)	1.5 (0.6, 2.5)
	44	3099	- #	0.6 (−0.4, 1.6)	0.9 (−0.2, 2.0)	1.9 (1.0, 2.9)	2.1 (1.1, 3.2)	1.1 (0.0, 2.3)
	50	2,759	- #	0.4 (−0.6, 1.5)	1.0 (−0.1, 2.1)	2.0 (1.0, 3.0)	2.2 (1.1, 3.3)	0.7 (−0.5, 1.9)
1970 BCS	26	3,545	-	0.6 (0.0, 1.2)	0.4 (−0.3, 1.1)	1.2 (0.6, 1.8)	1.3 (0.6, 1.9)	1.5 (0.7, 2.2)
	30	4,132	-	0.7 (0.0, 1.4)	0.7 (0.0, 1.5)	1.6 (1.0, 2.3)	1.8 (1.0, 2.5)	2.2 (1.4, 3.1)
	34	3,642	-	0.3 (−0.4, 1.1)	0.4 (−0.4, 1.3)	1.3 (0.6, 2.1)	1.4 (0.6, 2.3)	2.7 (1.7, 3.7)
	42	3,312	- #	0.3 (−0.6, 1.2)	0.1 (−0.9, 1.2)	1.7 (0.9, 2.6)	1.6 (0.6, 2.6)	2.7 (1.6, 3.9)

#evidence for deviation from linearity—*p* < 0.05 for Wald tests; BMI differences estimated using linear regression models; occupational-based social classes were assigned as follows: I (professional), II (managerial and technical), III NM (skilled nonmanual), III M (skilled manual), IV (partly-skilled) V (unskilled)

**Table 2:** Own social class (42/43 y) and BMI across mid-adulthood in the 1946 NSHD, 1958 NCDS, and 1970 BCS British birth cohort studies

			kg/m^2^ BMI difference (95% CI)					
	*Gender*, *age*							
Cohort	Men	N	I (ref)	II	III NM	III M	IV	V
1946 NSHD	43	1,520	- #	0.8 (0.1, 1.4)	1.0 (0.1, 1.9)	1.1 (0.4, 1.8)	0.5 (−0.4, 1.4)	0.0 (−1.5, 1.5)
	53	1,283	-	0.3 (−0.5, 1.1)	0.3 (−0.9, 1.5)	0.8 (−0.1, 1.6)	0.0 (−1.1, 1.2)	−0.9 (−2.5, 0.7)
	60–64	963	-	0.2 (−0.8, 1.1)	0.7 (−0.6, 2.0)	1.3 (0.3, 2.3)	0.4 (−1.0, 1.8)	0.5 (−3.6, 4.6)
1958 NCDS	42	4,623	-	0.7 (0.3, 1.2)	0.9 (0.4, 1.4)	0.9 (0.4, 1.3)	1.0 (0.4, 1.5)	0.8 (0.0, 1.5)
	44	3,808	- #	0.7 (0.2, 1.2)	0.8 (0.1, 1.4)	1.0 (0.5, 1.5)	1.3 (0.6, 2.0)	0.3 (−0.7, 1.3)
	50	3,283	-	0.9 (0.4, 1.5)	1.2 (0.5, 1.9)	1.6 (1.0, 2.2)	1.9 (1.2, 2.7)	1.2 (0.0, 2.4)
1970 BCS	42	3,636	-#	0.8 (0.2, 1.4)	1.0 (0.3, 1.8)	1.4 (0.8, 2.0)	1.0 (0.3, 1.7)	0.4 (−0.7, 1.5)
	Women							
1946 NSHD	43	1,419	-	−0.5 (−2.0, 1.1)	0.1 (−1.5, 1.6)	0.6 (−1.3, 2.6)	1.6 (−0.1, 3.4)	2.0 (−0.1, 4.0)
	53	1,264	-	0.0 (−2.4, 2.4)	0.4 (−1.9, 2.8)	0.5 (−2.2, 3.2)	1.9 (−0.6, 4.4)	2.3 (−0.5, 5.1)
	60–64	999	-	−0.1 (−2.2, 2.0)	0.6 (−1.5, 2.7)	1.3 (−1.1, 3.7)	1.5 (−0.8, 3.8)	1.9 (−1.1, 4.9)
1958 NCDS	42	4,139	- #	1.0 (0.0, 1.9)	0.8 (−0.1, 1.7)	1.8 (0.8, 2.9)	1.4 (0.5, 2.4)	2.3 (1.1, 3.4)
	44	3,421	- #	0.9 (−0.2, 2.0)	0.6 (−0.5, 1.7)	1.9 (0.7, 3.2)	1.5 (0.4, 2.6)	2.1 (0.7, 3.5)
	50	2,958	-	0.6 (−0.5, 1.7)	0.7 (−0.4, 1.8)	1.8 (0.5, 3.1)	1.3 (0.2, 2.5)	2.7 (1.2, 4.2)
1970 BCS	42	3,386	- #	1.5 (0.7, 2.4)	2.1 (1.2, 3.0)	2.8 (1.7, 3.9)	2.6 (1.7, 3.6)	3.9 (2.3, 5.4)

#evidence for deviation from linearity—*p* < 0.05 for Wald tests; BMI differences estimated using linear regression models; occupational-based social classes were assigned as follows: I (professional), II (managerial and technical), III NM (skilled nonmanual), III M (skilled manual), IV (partly-skilled) V (unskilled)

### BMI measurement

BMI (kg/m^2^), the main outcome measure, was derived in each study from measured or self-reported weight and height obtained at all available adulthood ages: 1946 NSHD: 20*, 26*, 36, 43, 53, and 60–64 y; 1958 NCDS: 23*, 33, 42*, 44, and 50* y; 1970 BCS: 26*, 30*, 34*, and 42* y (*self-report). The main protocol differences and methods used to harmonize height and weight have been described elsewhere.[6] Briefly, all measures were converted to metric units, women were excluded when pregnant (*N* = 257 (1946), 684 (1958), 110 (1970)), and a standardized cleaning process was used to remove participants with implausible values.

### SEP ascertainment

Indicators of SEP in childhood and adulthood were derived in each cohort—the main exposures in this study. Social class measures were used, since comparable measures were available in both childhood and adulthood, and in each cohort. Childhood SEP was indicated by father’s occupational social class, measured at 10/11 y, and adult SEP by own occupational social class measured at 42/43 y. To aid comparability of results across cohorts, the Registrar General’s Social Class was used to classify social class—from I (professional), II (managerial and technical), IIIN (skilled nonmanual), IIIM (skilled manual), IV (partly-skilled), and V (unskilled) occupations. The 1990 classification schema was used for childhood and adult SEP in all cohorts, and (due to a lack of conversion schema) the 1970 version was used for childhood SEP in the 1946 NSHD. Those in the armed forces were not assigned a social class, nor those not employed.

### Analytical strategy

DB, RH, WJ, LL, and DK determined which analyses to perform and include in the paper in August 2016. Following request from peer review, we conducted additional analyses in October 2016 to examine the extent to which associations between childhood SEP and BMI were explained by adult SEP, and to examine in greater detail the characteristics of those with missing data.

#### SEP differences in BMI

We derived mean BMI (and standard error [SE]) at each age for each childhood and adult SEP group, separately for each cohort and gender. To assess absolute inequalities in BMI at each age for childhood and adult SEP, we applied linear regression models to estimate differences in mean BMI (kg/m^2^) between each SEP class and the referent group (class I). We additionally checked if results differed when using regression models with log-transformed BMI to estimate relative differences (i.e., percentage) in BMI. To limit the potential for reverse causality, analyses using adult SEP were limited to contemporaneous and future BMI (≥42 y). Given expected gender differences in association (with larger inequalities in women),[7, 8] all analyses were conducted separately in each gender, and gender differences were formally tested by including an interaction term (gender*SEP). Deviation from linearity in the association between SEP and BMI was examined using Wald tests to determine whether coefficients for SEP (modelled as a categorical term) were equal to zero, in a model which also contained SEP modelled as a continuous term. To examine if the size of inequalities in BMI differed by cohort, regression models were also fitted on BMI at 42/43 y for all three cohorts combined, where differences in age of BMI measurements were smallest so that inequalities could be compared with respect to both childhood and adult SEP. A dummy term for each cohort was included in the model, and SEP*cohort interaction was tested. In all cohort-combined models, weights to account for the stratified nature of the 1946 NSHD cohort were applied, and all participants from 1958 NCDS and 1970 BCS were given the same weighting value of one. All analyses were conducted using statistical software STATA 14 (StataCorp, 2009).

#### SEP differences in risk of overweight or obesity

The primary analyses described above used BMI as a continuous outcome, since preservation of the continuous nature of the outcome preserves statistical power and may enable a greater understanding of the nature of adiposity inequalities than analyses using binary outcomes. However, to aid public health interpretation, all analyses were repeated using a binary outcome indicating normal (BMI < 25) or overweight/obese (BMI ≥ 25) as an outcome. Overweight and obese were grouped together given the low obesity prevalence at younger ages, and participants classified as thin were excluded from analyses (2% of observations).[6] Inequalities were estimated using linear probability models to derive differences in prevalence by SEP group, and using log-binomial generalized linear models to estimate the relative risks of overweight/obesity in each SEP group.

#### Do SEP differences in BMI differ by age?

Trajectories of BMI were modelled using multilevel models—BMI measurements (level 1) were nested within individuals (level 2). We adopted a quadratic function for age to summarize the longitudinal changes of BMI. We specified a random intercept and random slope (linear term for age). SEP (as a categorical variable) was added to the models to examine its associations with BMI across adulthood. Differences in rate of BMI change between SEP groups were examined by including age*SEP interaction terms (age^2^*SEP interactions were not found and therefore not included in models; *p* > 0.05). Finally, a cohort combining all three cohorts was fitted, and an age*SEP*cohort interaction term was included to test whether the change in association by age differed by cohort. This model also contained cohort main effects, and all two-way interactions (age*SEP, age*cohort, SEP*cohort). Only fixed effects models were fitted when analysing adult SEP in 1970 BCS, since only one age point of BMI was available. Finally, additional models were conducted to examine whether associations between childhood SEP and BMI were explained by adult SEP. In these models, participants with valid data for SEP and valid BMI data on at least one age were included in analyses.

#### Additional and sensitivity analyses

To examine the extent to which self-reported BMI data could bias SEP and BMI associations, we calculated differences in BMI in the 1958 NCDS at 42 y (self-reported) and 44 y (objectively measured)—the closest proximity of BMI self-reporting and objective measures in our data. We then examined relations between SEP and this difference measure: larger scores would indicate misreporting and/or excessive weight change. We were unable to adjust for an indicator of self-reporting or objective BMI measurement method in our models due to collinearity between measurement method and age/cohort. Finally, to inform the extent to which differences in missing data between cohorts could affect results, we compared the extent of missing SEP and BMI data by cohort and examined the characteristics of those with missing data.

## Results

BMI was typically higher at older ages within each cohort and in cohorts born more recently. BMI was also generally higher amongst those of lower rather than higher SEP (S1 and S2 Tables). Obesity or overweight prevalence followed the same patterns (S3 and S4 Tables).

### Childhood SEP

In each cohort, lower childhood SEP was associated with higher mean BMI at all ages (Table 1). Associations tended to be stronger among women than men: evidence for gender interaction with childhood SEP (P<0.05) was found in the 1946 NSHD (at 60–64 y), the 1958 NCDS (33, 42, 44, and 50 y), and the 1970 BCS (30 and 42 y). Associations were also found to be nonlinear in many cases, with kg/m^2^ differences in BMI between classes I and IV (partly skilled) being larger than those between I and V (unskilled).

The sizes of SEP differences in BMI were of considerable magnitude. For example, comparing mean BMI at 42/43 y amongst women in the lowest compared with highest childhood SEP, there was a 1.7 kg/m^2^ (95% CI: 0.2, 3.2) difference in the 1946 NSHD, 1.5 kg/m^2^ (0.6, 2.5) difference in the 1958 NCDS, and 2.7 kg/m^2^ (1.6, 3.9) difference in the 1970 BCS. However, there was little evidence that the size of BMI inequalities differed systematically by cohort (*p*-value for cohort*childhood SEP term = 0.33 in men and 0.20 in women; findings were similar when analyzing BMI differences in relative instead of absolute scales). Analyses using overweight or obese as a binary outcome yielded similar results (S5 Table). For example, comparing the difference in prevalence at 42/43 y amongst women in the lowest compared with highest childhood SEP, there was a 22.5% (5.9%, 39.2%) difference in the 1946 NSHD, 14.2% (5.1%, 23.3%) in the 1958 NCDS, and 15.7% (5.4%, 25.9%) in the 1970 BCS.

**Fig 1:**
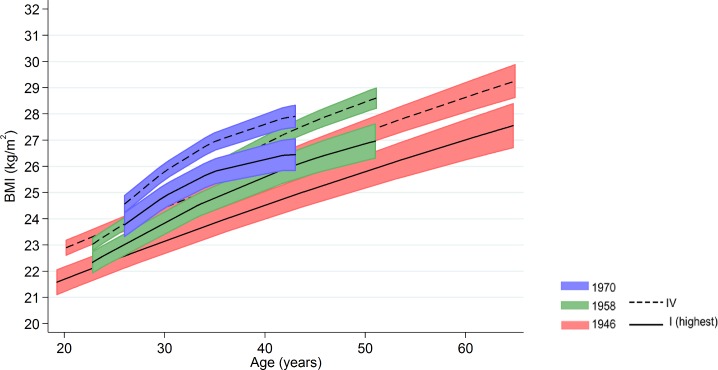
Male BMI across adulthood in relation to father’s social class (10/11 y) in the 1946, 1958, and 1970 British birth cohort studies. Note: lines show estimated BMI along with 95% confidence intervals at each age, estimated using multilevel general linear regression models.

**Fig 2:**
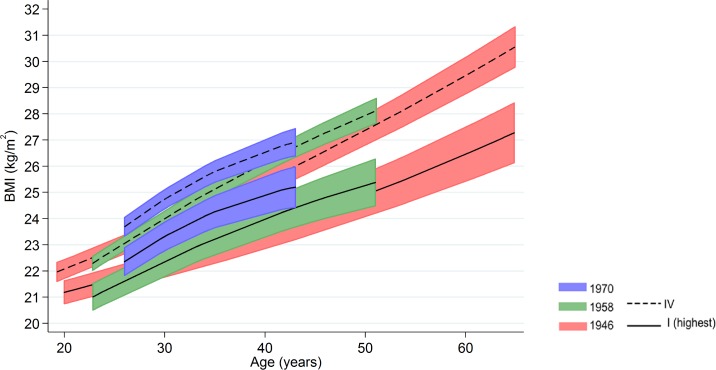
Female BMI across adulthood in relation to father’s social class (10/11 y) in the 1946, 1958, and 1970 British birth cohort studies. Note: lines show estimated BMI along with 95% confidence intervals at each age, estimated using multilevel general linear regression models.

Inequalities in BMI within each cohort were typically larger at older ages, as indicated by positive age*SEP interaction terms for the lower SEP groups (Figs 1 and 2, multilevel model estimates shown in S6 Table). For example, among women in the 1946 NSHD, the estimated BMI difference between class I and V of 1.45 kg/m^2^ (0.61, 2.29) at 26 years had increased to 3.30 kg/m^2^ (1.38, 5.21) at 60–64 y. The size of these age-related increases in inequality did not appear to differ systematically by cohort except in men, where age-related increases appeared to be stronger in the 1958 NCDS and 1970 BCS than the 1946 NSHD (*p* = 0.001 age*SEP*cohort interaction; *p* = 0.01 in women). These associations occurred alongside both age-related increases in mean BMI and secular increases in mean BMI in later born cohorts—mean BMI in the highest SEP group in the BCS 1970 was comparable to that of the lowest SEP group in the 1946 NSHD. Finally, associations between childhood SEP and BMI were typically only partly attenuated after adjustment for adult SEP (S7 Table).

### Adult SEP

Lower adult SEP was typically associated with higher BMI (Table 2). As with childhood SEP differences, these associations were typically stronger among women than men—evidence for gender interaction with adult SEP (*p* < 0.05) was found in the 1946 NSHD (at 43, 53, and 60–64 y), the 1958 NCDS (42 and 44 y), and the 1970 BCS (42 y)—and for nonlinearity in the shape of associations.

Among women, the size of BMI inequalities was progressively larger in each subsequent cohort. For example, the mean differences in BMI at 42/43 y amongst women in the lowest compared with highest social class was 2.0 kg/m^2^ (95% CI: −0.1, 4.0) in the 1946 NSHD, 2.3 (1.1, 3.4) in the 1958 NCDS, and 3.9 (2.3, 5.4) in the 1970 BCS—*p*(for cohort*SEP term) = 0.01. These cohort differences were driven by slight decreases in mean BMI in the highest social class and increases in BMI amongst those in the lowest social classes (S2 Table). There was no such evidence of cohort differences in men (*p* = 0.7), and findings for either gender did not differ when using relative instead of absolute measures of inequality. For example, among women, percentage differences in BMI at 42/43 in the lowest compared with highest social class were 6.4% (−1.3, 14.1) in the 1946 NSHD, 8.1% (4.0, 12.3) in the 1958 NCDS, and 14.0% (8.5, 19.6) in 1970 BCS. Mean (standard deviation [SD]) BMI in the highest and lowest social classes were as follows: 24.9 (0.8) versus 26.8 (0.7) in the 1946 NSHD, 24.2 (0.4) versus 26.5 (0.4) in the 1958 NCDS, and 24.2 (0.3) versus 28.1 (0.8) in the 1970 BCS (see S2 Table). Analyses using overweight or obese as binary outcomes yielded similar results (S8 Table). For example, when comparing the percentage difference in prevalence at 42/43 y amongst women in the lowest compared with highest adult SEP, there was a 5.2% (−26.2, 36.5) difference in the 1946 NSHD, 20.2% (8.6, 31.9) in the 1958 NCDS, and 26.9% (12.5, 41.2) in the 1970 BCS.

**Fig 3:**
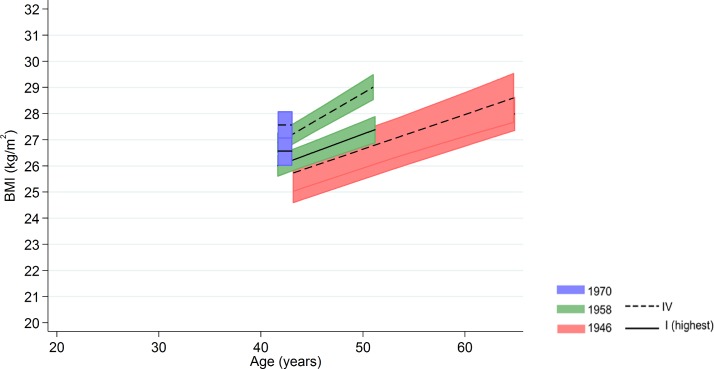
Male BMI across adulthood in relation to own social class (42/43 y) in the 1946 NSHD, 1958 NCDS, and 1970 BCS British birth cohort studies. Note: lines show estimated BMI along with 95% confidence intervals at each age, estimated using multilevel general linear regression models (age terms not included in the 1970 BCS due to only 1 age of measurement).

**Fig 4:**
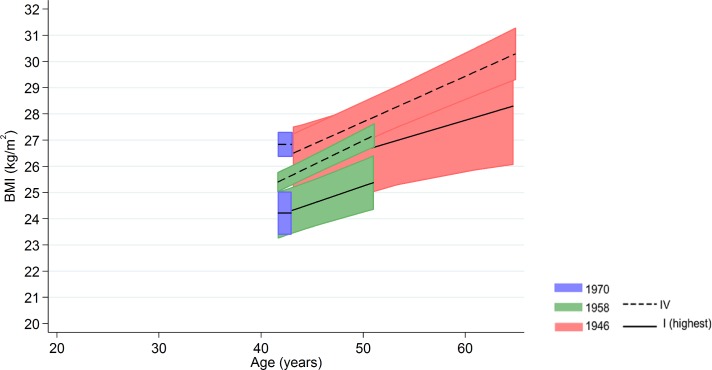
Female BMI across adulthood in relation to own social class (42/43 y) in the 1946 NSHD, 1958 NCDS, and 1970 BCS British birth cohort studies. Note: lines show estimated BMI along with 95% confidence intervals at each age, estimated using multilevel general linear regression models (age terms not included in the 1970 BCS due to only one age of measurement).

Among men in the 1958 NCDS, adult SEP and BMI associations tended to become larger at older ages—the estimated difference in BMI between class IV and I was 0.98 kg/m^2^ (0.43, 1.54) at 43 y and 1.57 kg/m^2^ (0.91, 2.23) at 50 y (Fig 3 and Fig 4 and S9 Table). These differences were not found among men in the 1946 NSHD (*p* < 0.001 age*SEP*cohort interaction). Among women, there was no evidence that associations systematically changed with age in either cohort (*p* = 0.97 age*SEP*cohort interaction).

### Additional and sensitivity analyses

We did not find evidence that differences in BMI at 42 (self-reported) and 44 y (objectively measured) differed according to childhood (*p* = 0.9 in men, *p* = 0.2 in women) or adult SEP (*p* = 0.3 in men, *p* = 0.3 in women) in the 1958 NCDS (S10 Table). More recently born cohorts had greater missing SEP and BMI data; similarly in each cohort, those with lower childhood SEP or higher preceding BMI were more likely to have missing adult SEP and BMI data (S1 Text).

## Discussion

### Main findings

Using longitudinal data from three British birth cohorts who experienced the obesity epidemic at increasingly younger ages in adulthood, we identified large and persisting socioeconomic inequalities in BMI from age 20 up to ages 60–64. Inequalities according to childhood SEP were evident in both men and women, were typically larger at older ages, and were similar in magnitude at any given age in all three cohorts. Inequalities according to adult SEP were more evident in women than men, yet did not consistently differ by age. Among women, inequalities were found to be larger in both absolute and relative terms in the 1970 BCS compared with earlier-born cohorts in midlife BMI (i.e., BMI at age 42/43 y when measured in 2012 compared with 2000 or 1989), while in men they remained constant.

### Comparison with previous studies

Our findings are broadly consistent with evidence of persisting inequalities in obesity or BMI derived from separate studies utilizing repeated cross-sectional data and adult SEP indicators. For example, in English adults aged 18–64, BMI inequalities according to adult manual or nonmanual social class did not change (in absolute terms) from 1993/4 to 2002/3,[13] yet obesity inequalities increased in absolute but not relative terms according to area-based socioeconomic circumstances among older (age ≥55) but not younger (16–54) adults from 1994 to 2008.[14] In Scotland, obesity inequalities (in relative terms) according to education or income in adults were estimated to have decreased from 1995 to 2011 (age 16–65).[15] Studies using birth cohort data have also reported inequalities in BMI yet are either limited to investigation of single birth cohorts,[22–25, 27] or use multiple birth cohorts in a more limited manner (e.g., among regional samples with limited overlap between ages/y born,[47] or among two national birth cohorts using BMI at one age and a binary SEP indicator[26]). Our findings add to the international evidence base, also derived from repeated cross-sectional data, suggesting persisting inequalities in high income countries and/or emerging inequalities in low–middle income countries.[17–21]

Our study adds to previous knowledge by providing a more detailed understanding of how inequalities in BMI have changed across a longer timeframe. Despite large societal changes in occupations and a reduction in absolute disadvantage in the later 20th century, we found that social class inequalities have persisted across three generations. Two additional findings also suggest a greater potential cause for concern than previously thought: for childhood SEP, associations typically widened with increasing age in all three cohorts and, for adult SEP, associations appear to have become larger in more recently born generations of women.

### Explanation of findings

Inequalities in BMI are likely to be explained by inequalities in the determinants of weight gain both before and during adulthood. In support of this, systematic reviews and large population-based studies have found evidence for inequalities in both indicators of physical activity[48, 49] and diet.[50–52] The persistence of inequalities in BMI across cohorts suggests that inequalities in the determinants of BMI have not substantially changed,[53] despite policies designed to reduce them.[54, 55] The net influence of childhood SEP on the determinants of BMI, operating both in childhood and adulthood, may therefore have not differed in each cohort. If this were the case, our finding of increasing size of inequalities in BMI found at older compared with younger ages (according to childhood SEP) may be due to the accumulation of periods of weight gain which would be expected to track across life.[56] Part of the childhood SEP and BMI associations are also likely to be explained by continuity of SEP into adulthood, although we found limited evidence for a prominent mediating role of adult social class (as in other studies[22–26]). However, such pathways may be more appropriately identified in analyses that account for potential intermediary confounding[57] and the multidimensional and time-varying nature of adult SEP.

Although we are not aware of studies that have examined trends in diet and physical activity inequalities in these birth cohorts, there are some co-occurring cultural and economic changes which may have been expected to have increased BMI inequalities across time. For example, the relative cost of a high-quality diet increased in the UK (and other high income countries) from the 1990s–2010,[58] although it is unclear if this was matched by increased inequalities in dietary intake.[14, 59] In addition, studies have suggested that there have been increasing cultural expectations from the 1970s onwards for women to be thin,[60] yet for men to be muscular.[61, 62] Since women of higher SEP are likely to have had greater resources to follow these trends, these changes could explain the increasing inequalities observed in the 1970 BCS compared with earlier born cohorts; mean BMI in the most advantaged group remained similar in all three cohorts. Women also experience additional putative risk factors for weight gain which men do not, such as childbirth and the menopausal transition,[63] yet these factors have not explained BMI inequalities in previous studies.[64] Inequalities in leisure time physical activity widened among older women but not men in England from 1994–2008 (according to an area-based socioeconomic indicator)[14], and occupational-related physical activity may have declined, especially amongst manual social classes, due to declines in the number of physical occupations.[65] However, it should be noted that understanding the relative contribution of diet and physical activity to BMI inequalities, and how these may change across time, is likely to be challenging due to the difficulties in accurately assessing these behaviours in the population.

In addition, labour market participation has changed across time, with increasing participation from women in the 1970 BCS compared with the 1946 NSHD.[66] Such differences could potentially affect estimates of how inequalities in BMI have changed across time, particularly among women. A conservative interpretation of our results would therefore limit them to providing evidence of how socioeconomic inequality in BMI has changed amongst those whose fathers were employed as children, or those subsequently employed as adults. Despite overall declining population smoking rates, relative inequalities in smoking have increased,[67] a pattern which could affect BMI inequality, since smoking has a modest association with lower weight. However, smoking has been found not to explain associations between SEP and BMI in other cohorts,[68] and smoking was not associated with obesity in the 1946 NSHD,[69] nor in the 1970 BCS.[70] Finally, mortality rates are likely to have been highest in lower SEP groups[71] and those with higher BMI,[72] which might partly explain the lower-than-expected BMI found in the lowest SEP group among men at older ages.

### Strengths and limitations

Strengths of this study include the use of three national birth cohort studies initiated in 1946, 1958, and 1970, with harmonized data for SEP and BMI across life. These data are especially well-suited to examine the emergence of inequalities in BMI across age and generation. Limitations include the use of BMI which, although strongly positively correlated with fat mass[73], does not distinguish fat and lean (muscle) mass. Lower SEP has been associated with lower lean mass in the 1946 NSHD (after adjustment)[23], so the use of BMI could have resulted in underestimating socioeconomic inequalities in fat mass, compared with direct measures of fat mass. Indeed, given evidence that BMI and blood pressure associations are stronger in the 1958 NCDS than the 1946 NSHD,[74] it may be amongst later born cohorts a given BMI value reflects increasingly more fat than lean mass compared with earlier born cohorts.

All included studies experienced attrition that, as expected,[43, 44] was generally more pronounced amongst those of lower SEP and/or with higher BMI. The use of multilevel models enabled those with incomplete information to be included in our analyses (under the assumption of missing at random). However, as in all observational studies, we cannot rule out the possibility that missing data are nonignorable and therefore might bias our findings. Missing SEP and BMI data were also more frequent in later born cohorts, which might bias results regarding cross cohort changes in BMI inequality. Studies have found that greater attrition or nonresponse in longitudinal studies typically leads to a reduction in the magnitude of health inequalities observed;[75] analogous findings were also found in the present study, since childhood SEP and BMI associations were generally weaker in the samples which additionally had valid adult SEP data (S7 Table). This direction of bias may therefore have led to us underestimating increases in BMI inequality observed amongst more recently born cohorts (which were observed for women), and increases in the size of BMI inequality at older ages (which were found for both men and women). In support of the representativeness of the included birth cohorts, obesity estimates have been found to be similar with those from the Health Survey for England.[6] However, such biases should be considered when interpreting these, and other, results on how SEP differences may have changed across time—cross-sectional health surveys have moderate response rates (around 40 to 60%) which have declined in recent decades.[76] We used harmonized measures of occupational-based social class, yet these cohorts predate the 2000 move to NS-SEC social class used in the UK.[77] The use of social class omitted participants not currently employed; class is also only one dimension of SEP—others (e.g., education, income, and wealth) may yield health-relevant information independent of class, and therefore warrant further study. Finally, we focused on average differences across social groups, whereas within-group differences may have also changed by cohort, and this warrants investigation.[78]

### Implications

The persistence of inequalities in BMI throughout adulthood across different generations suggests that new and/or improved strategies are required to reduce them. Indeed, the UK government has, through a number of initiatives, aimed to reduce both obesity and its inequality since 1998, with limited current success.[54, 55] While there is some evidence that interventions targeted at disadvantaged populations can result in reductions in BMI, there is limited high-quality evidence on population-wide interventions, as well as on the relative timing of interventions across life.[79] Given our findings of progressively widening BMI inequalities across adulthood, and the fact that BMI tends to track across life,[56] interventions may be most effective when initiated as early as possible in adulthood. Interventions which require little individual agency[80] may be especially efficacious at lowering population BMI levels and reducing inequalities. For example, increasing taxation on unhealthy foods while subsidizing others may be effective, despite potentially being financially regressive in the short term.[81, 82] Coordinated analyses of how inequalities in BMI differ across countries may also help to identify strategies which successfully reduce BMI inequalities.[83] Ultimately, targeting inequalities in socioeconomic resources may be more effective than targeting specific mediating factors, since the relative importance of the mediators may change across time and interventions may inadvertently increase inequalities.[84] Reducing inequalities in socioeconomic resources in childhood may be particularly beneficial by affecting both early life determinants of adult BMI and subsequent adult SEP inequalities. Indeed, such early interventions will also be best placed to reduce inequalities in childhood obesity, which are found in cohorts born more recently (e.g., those born in 2000/1);[85] understanding how adiposity inequalities have changed in different generations in childhood requires investigation of childhood growth in terms of weight, height, and height-adjusted weight.

## Conclusions

Our findings, based on historic longitudinal data, demonstrate that the overweight and obesity epidemic has disproportionately impacted adults in Britain born in 1946, 1958, and 1970 who were more socioeconomically disadvantaged in childhood or adulthood. They prompt consideration of how inequalities can be reduced amongst these and future cohorts, given the considerable expected adverse health impacts.

## Supporting Information

S1 Table Father’s occupational class (10/11 y) and mean BMI across adulthood in the 1946 NSHD, 1958 NCDS, and 1970 BCS British birth cohort studies. (DOC)

S2 Table Own occupational class (42/43 y) and mean BMI across adulthood in the 1946 NSHD, 1958 NCDS, and 1970 BCS British birth cohort studies. (DOC)

S3 Table Father’s occupational class (10/11 y) and overweight or obesity prevalence across adulthood in the 1946 NSHD, 1958 NCDS, and 1970 BCS British birth cohort studies. (DOC)

S4 Table Own occupational class (42/43 y) and overweight or obesity prevalence across adulthood in the 1946 NSHD, 1958 NCDS, and 1970 BCS British birth cohort studies. (DOC)

S5 Table Father’s occupational class (10/11 y) and obesity or overweight across adulthood in the 1946 NSHD, 1958 NCDS, and 1970 BCS British birth cohort studies. (DOC)

S6 Table Father’s occupational class (10/11 y) and BMI across adulthood (≥20 y) in the 1946 NSHD, 1958 NCDS, and 1970 BCS British birth cohort studies: estimates from separate multilevel models, scaled to show estimated BMI differences at 26 y. (DOC)

S7 Table Father’s occupational class (10/11 y) and BMI across adulthood (≥20 y) in the 1946 NSHD, 1958 NCDS, and 1970 BCS British birth cohort studies, adjusted for adult occupational class (42/43 y): estimates from separate multilevel models, scaled to show estimated BMI differences at 26 y. (DOC)

S8 Table Own occupational class (42/43 y) and obesity or overweight across mid-adulthood in the 1946 NSHD, 1958 NCDS, and 1970 BCS birth cohort studies. (DOC)

S9 Table Own occupational class (42/43 y) and adult BMI (≥42 y) in the 1946 NSHD and 1958 NCDS British birth cohort studies: estimates from separate multilevel models, scaled to show estimated BMI differences at 43 y. (DOC)

S10 Table Socioeconomic position in relation to calculated change in BMI between a self-reported (at 42 y) and objective measure (at 44 y) in the 1958 NCDS British birth cohort study (DOC)

S1 PRIMSA Checklist (DOC)

S1 Text Missing data appendix. (DOC)

## Abbreviations

BMI: body mass index
SD: standard deviation
SE: standard error
SEP: socioeconomic position
