# Supplementary material for: Socioeconomic Inequalities in Body Mass Index across Adulthood: Coordinated Analyses of Individual Participant Data from Three British Birth Cohort Studies Initiated in 1946, 1958 and 1970
Source: PLoS Med. 2017 Jan 10;14(1):e1002214. doi: 10.1371/journal.pmed.1002214 (PMC5224787; doi:10.1371/journal.pmed.1002214)
Supplement: S1 Table — (DOC) [file pmed.1002214.s001.doc]

S1 Table. Father’s occupational class (10/11y) and mean body mass index across adulthood in the 1946 NSHD, 1958 NCDS, and 1970 BCS British birth cohort studies

|  |  | | |  |  | | | Father’s occupational class (10/11y),  BMI, mean (SE) | | | |  |
| --- | --- | --- | --- | --- | --- | --- | --- | --- | --- | --- | --- | --- |
| Cohort | *Gender, age*  Men | N | I | | | II | III NM | | III M | IV | V |  |
| 1946 NSHD | 20 | 1562 | 22.5 (0.2) | | | 22.5 (0.2) | 22.2 (0.2) | | 22.8 (0.1) | 22.8 (0.2) | 23.3 (0.3) |  |
|  | 26 | 1552 | 23.2 (0.2) | | | 23.2 (0.2) | 23.0 (0.2) | | 23.6 (0.1) | 23.8 (0.2) | 24.2 (0.3) |  |
|  | 36 | 1365 | 24.6 (0.2) | | | 24.6 (0.2) | 24.3 (0.2) | | 25.2 (0.2) | 25.4 (0.2) | 25.1 (0.4) |  |
|  | 43 | 1338 | 25.4 (0.2) | | | 25.4 (0.2) | 25.3 (0.3) | | 26.2 (0.2) | 26.4 (0.3) | 25.8 (0.4) |  |
|  | 53 | 1206 | 27.0 (0.3) | | | 27.0 (0.3) | 26.7 (0.3) | | 27.9 (0.2) | 28.2 (0.3) | 27.5 (0.5) |  |
|  | 60-64 | 898 | 27.4 (0.4) | | | 27.4 (0.4) | 27.2 (0.3) | | 28.8 (0.3) | 28.8 (0.4) | 27.7 (0.7) |  |
|  |  |  |  | | |  |  | |  |  |  |  |
| 1958 NCDS | 23 | 4046 | 22.8 (0.1) | | | 22.8 (0.1) | 22.8 (0.1) | | 23.3 (0.1) | 23.2 (0.1) | 23.3 (0.2) |  |
|  | 33 | 3547 | 25.2 (0.1) | | | 25.2 (0.1) | 25.5 (0.2) | | 25.7 (0.1) | 25.9 (0.2) | 25.7 (0.2) |  |
|  | 42 | 3637 | 26.2 (0.1) | | | 26.2 (0.1) | 26.2 (0.2) | | 26.5 (0.1) | 26.7 (0.2) | 27.0 (0.2) |  |
|  | 44 | 3046 | 27.4 (0.2) | | | 27.4 (0.2) | 27.6 (0.2) | | 27.9 (0.1) | 28.3 (0.2) | 28.5 (0.3) |  |
|  | 50 | 2766 | 27.5 (0.2) | | | 27.5 (0.2) | 27.7 (0.2) | | 28.2 (0.1) | 28.8 (0.3) | 28.7 (0.3) |  |
|  |  |  |  | | |  |  | |  |  |  |  |
| 1970 BCS | 26 | 1923 | 24.1 (0.2) | | | 24.1 (0.2) | 23.8 (0.2) | | 24.6 (0.1) | 25.0 (0.2) | 25.0 (0.4) |  |
|  | 30 | 3960 | 25.4 (0.1) | | | 25.4 (0.1) | 25.2 (0.2) | | 25.8 (0.1) | 25.6 (0.2) | 25.6 (0.2) |  |
|  | 34 | 3435 | 26.2 (0.1) | | | 26.2 (0.1) | 26.2 (0.2) | | 26.8 (0.1) | 26.8 (0.2) | 26.9 (0.3) |  |
|  | 42 | 3208 | 26.9 (0.1) | | | 26.9 (0.1) | 27.2 (0.3) | | 27.9 (0.1) | 27.9 (0.3) | 27.5 (0.3) |  |
|  |  |  |  | | |  |  | |  |  |  |  |

|  |  | | |  |  | | | Father’s occupational class (10/11y),  BMI, mean (SE) | | | |  |
| --- | --- | --- | --- | --- | --- | --- | --- | --- | --- | --- | --- | --- |
| Cohort | *Gender, age*  Women | N | I | | | II | III NM | | III M | IV | V |  |
| 1946 NSHD | 20 | 1426 | 21.8 (0.2) | | | 21.8 (0.2) | 21.3 (0.2) | | 21.8 (0.1) | 22.2 (0.2) | 22.4 (0.4) |  |
|  | 26 | 1543 | 22.2 (0.2) | | | 22.2 (0.2) | 22.0 (0.2) | | 22.6 (0.2) | 23.1 (0.2) | 23.4 (0.5) |  |
|  | 36 | 1353 | 23.1 (0.3) | | | 23.1 (0.3) | 22.5 (0.2) | | 23.9 (0.2) | 24.5 (0.3) | 24.3 (0.5) |  |
|  | 43 | 1315 | 24.6 (0.3) | | | 24.6 (0.3) | 24.3 (0.3) | | 25.5 (0.3) | 26.3 (0.4) | 25.8 (0.6) |  |
|  | 53 | 1235 | 27.0 (0.4) | | | 27.0 (0.4) | 26.6 (0.4) | | 27.8 (0.3) | 29.1 (0.5) | 27.7 (0.8) |  |
|  | 60-64 | 958 | 27.7 (0.5) | | | 27.7 (0.5) | 26.8 (0.4) | | 28.1 (0.3) | 30.2 (0.5) | 29.7 (1.2) |  |
|  |  |  |  | | |  |  | |  |  |  |  |
| 1958 NCDS | 23 | 4115 | 21.7 (0.1) | | | 21.7 (0.1) | 21.6 (0.1) | | 22.4 (0.1) | 22.4 (0.1) | 22.2 (0.2) |  |
|  | 33 | 3524 | 23.8 (0.2) | | | 23.8 (0.2) | 24.0 (0.2) | | 25.0 (0.1) | 25.0 (0.2) | 24.4 (0.3) |  |
|  | 42 | 3668 | 24.5 (0.2) | | | 24.5 (0.2) | 24.5 (0.2) | | 25.7 (0.1) | 26.0 (0.2) | 25.2 (0.3) |  |
|  | 44 | 3099 | 26.1 (0.2) | | | 26.1 (0.2) | 26.4 (0.3) | | 27.4 (0.2) | 27.6 (0.3) | 26.6 (0.4) |  |
|  | 50 | 2759 | 25.8 (0.2) | | | 25.8 (0.2) | 26.4 (0.3) | | 27.4 (0.2) | 27.5 (0.3) | 26.1 (0.4) |  |
|  |  |  |  | | |  |  | |  |  |  |  |
| 1970 BCS | 26 | 3545 | 22.9 (0.1) | | | 22.9 (0.1) | 22.7 (0.2) | | 23.5 (0.1) | 23.6 (0.2) | 23.8 (0.3) |  |
|  | 30 | 4132 | 23.7 (0.1) | | | 23.7 (0.1) | 23.8 (0.2) | | 24.6 (0.1) | 24.8 (0.2) | 25.2 (0.4) |  |
|  | 34 | 3642 | 24.6 (0.2) | | | 24.6 (0.2) | 24.7 (0.3) | | 25.6 (0.1) | 25.7 (0.3) | 26.9 (0.4) |  |
|  | 42 | 3312 | 25.5 (0.2) | | | 25.5 (0.2) | 25.3 (0.3) | | 26.9 (0.2) | 26.7 (0.3) | 27.9 (0.5) |  |
|  |  |  |  | | |  |  | |  |  |  |  |
