# Supplementary material for: Socioeconomic Inequalities in Body Mass Index across Adulthood: Coordinated Analyses of Individual Participant Data from Three British Birth Cohort Studies Initiated in 1946, 1958 and 1970
Source: PLoS Med. 2017 Jan 10;14(1):e1002214. doi: 10.1371/journal.pmed.1002214 (PMC5224787; doi:10.1371/journal.pmed.1002214)
Supplement: S2 Table — (DOC) [file pmed.1002214.s002.doc]

S2 Table. Own occupational class (42/43y) and mean body mass index across adulthood in the 1946 NSHD, 1958 NCDS, and 1970 BCS British birth cohort studies

|  |  | | |  | Own occupational class (42/43y),  BMI, mean (SE) | | | | | | |  |
| --- | --- | --- | --- | --- | --- | --- | --- | --- | --- | --- | --- | --- |
| Cohort | *Gender, age*  Men | N | I | | | II | III NM | III M | IV | V |  | |
| 1946 NSHD | 43 | 1520 | 25.1 (0.3) | | | 25.9 (0.2) | 26.1 (0.4) | 26.2 (0.2) | 25.7 (0.4) | 25.2 (0.7) |  | |
|  | 53 | 1283 | 27.2 (0.3) | | | 27.5 (0.2) | 27.5 (0.5) | 27.9 (0.2) | 27.2 (0.5) | 26.3 (0.7) |  | |
|  | 60-64 | 963 | 27.6 (0.4) | | | 27.7 (0.2) | 28.3 (0.5) | 28.9 (0.3) | 28.0 (0.6) | 28.1 (2.0) |  | |
|  |  |  |  | | |  |  |  |  |  |  | |
| 1958 NCDS | 42 | 4623 | 25.7 (0.2) | | | 26.4 (0.1) | 26.6 (0.2) | 26.6 (0.1) | 26.7 (0.2) | 26.5 (0.4) |  | |
|  | 44 | 3808 | 27.1 (0.2) | | | 27.8 (0.1) | 27.9 (0.2) | 28.1 (0.1) | 28.4 (0.3) | 27.4 (0.5) |  | |
|  | 50 | 3283 | 26.9 (0.2) | | | 27.9 (0.1) | 28.1 (0.2) | 28.5 (0.1) | 28.8 (0.3) | 28.1 (0.6) |  | |
|  |  |  |  | | |  |  |  |  |  |  | |
| 1970 BCS | 42 | 3636 | 26.6 (0.2) | | | 27.4 (0.1) | 27.6 (0.2) | 28.0 (0.1) | 27.6 (0.3) | 27.0 (0.6) |  | |
|  |  |  |  | | |  |  |  |  |  |  | |
|  | Women |  |  | | |  |  |  |  |  |  | |
| 1946 NSHD | 43 | 1419 | 24.9 (0.8) | | | 24.4 (0.2) | 24.9 (0.2) | 25.5 (0.6) | 26.5 (0.4) | 26.8 (0.7) |  | |
|  | 53 | 1264 | 26.9 (1.2) | | | 26.9 (0.3) | 27.4 (0.3) | 27.5 (0.7) | 28.9 (0.5) | 29.2 (0.8) |  | |
|  | 60-64 | 999 | 27.5 (1.0) | | | 27.5 (0.3) | 28.1 (0.3) | 28.8 (0.6) | 29.0 (0.6) | 29.5 (1.1) |  | |
|  |  |  |  | | |  |  |  |  |  |  | |
| 1958 NCDS | 42 | 4139 | 24.2 (0.4) | | | 25.1 (0.1) | 25.0 (0.1) | 26.0 (0.3) | 25.6 (0.2) | 26.5 (0.4) |  | |
|  | 44 | 3421 | 25.9 (0.5) | | | 26.8 (0.2) | 26.5 (0.1) | 27.9 (0.4) | 27.4 (0.2) | 28.0 (0.5) |  | |
|  | 50 | 2958 | 25.8 (0.5) | | | 26.5 (0.2) | 26.6 (0.2) | 27.7 (0.4) | 27.2 (0.3) | 28.5 (0.6) |  | |
|  |  |  |  | | |  |  |  |  |  |  | |
| 1970 BCS | 42 | 3386 | 24.2 (0.3) | | | 25.7 (0.1) | 26.3 (0.2) | 27.0 (0.4) | 26.8 (0.3) | 28.1 (0.8) |  | |
