# Supplementary material for: Socioeconomic Inequalities in Body Mass Index across Adulthood: Coordinated Analyses of Individual Participant Data from Three British Birth Cohort Studies Initiated in 1946, 1958 and 1970
Source: PLoS Med. 2017 Jan 10;14(1):e1002214. doi: 10.1371/journal.pmed.1002214 (PMC5224787; doi:10.1371/journal.pmed.1002214)
Supplement: S3 Table — (DOC) [file pmed.1002214.s003.doc]

S3 Table. Father’s occupational class (10/11y) and overweight or obesity prevalence across adulthood in the 1946 NSHD, 1958 NCDS, and 1970 BCS British birth cohort studies

|  |  | | |  |  | | | Father’s occupational class (10/11y),  Overweight or obese, % (SE) | | | |  |
| --- | --- | --- | --- | --- | --- | --- | --- | --- | --- | --- | --- | --- |
| Cohort | *Gender, age*  Men | N | I | | | II | III NM | | III M | IV | V |  |
| 1946 NSHD | 20 | 1523 | 8.0 (2.6) | | | 11.8 (2.3) | 12.7 (2.6) | | 15.1 (1.7) | 15.9 (2.7) | 23.7 (4.9) |  |
|  | 26 | 1520 | 13.6 (3.3) | | | 21.9 (3.0) | 20.8 (3.4) | | 30.2 (2.2) | 30.5 (3.3) | 30.1 (5.0) |  |
|  | 36 | 1346 | 28.1 (5.8) | | | 41.4 (3.7) | 36.0 (4.0) | | 51.8 (2.6) | 54.9 (3.7) | 46.2 (6.0) |  |
|  | 43 | 1328 | 41.0 (6.4) | | | 55.2 (3.7) | 50.4 (4.1) | | 64.9 (2.5) | 60.3 (3.8) | 57.4 (6.0) |  |
|  | 53 | 1202 | 67.4 (6.2) | | | 68.9 (3.5) | 65.9 (4.0) | | 80.1 (2.2) | 78.1 (3.2) | 73.1 (5.3) |  |
|  | 60-64 | 896 | 70.5 (6.5) | | | 73.5 (3.8) | 67.7 (4.6) | | 87.1 (2.2) | 80.2 (3.7) | 61.5 (7.3) |  |
|  |  |  |  | | |  |  | |  |  |  |  |
| 1958 NCDS | 23 | 3954 | 10.8 (2.3) | | | 16.9 (1.3) | 15.7 (1.8) | | 22.6 (1.0) | 21.1 (1.8) | 22.9 (2.3) |  |
|  | 33 | 3508 | 44.0 (3.8) | | | 47.6 (1.8) | 50.5 (2.6) | | 52.7 (1.3) | 55.8 (2.3) | 57.8 (2.9) |  |
|  | 42 | 3622 | 56.7 (3.7) | | | 60.4 (1.8) | 62.3 (2.4) | | 62.2 (1.3) | 63.1 (2.2) | 69.7 (2.6) |  |
|  | 44 | 3039 | 68.8 (3.7) | | | 71.9 (1.8) | 74.0 (2.4) | | 75.3 (1.2) | 79.4 (2.0) | 78.4 (2.6) |  |
|  | 50 | 2758 | 68.8 (3.9) | | | 70.2 (1.9) | 71.4 (2.6) | | 75.8 (1.3) | 78.6 (2.2) | 80.7 (2.6) |  |
|  |  |  |  | | |  |  | |  |  |  |  |
| 1970 BCS | 26 | 1901 | 27.4 (4.1) | | | 33.6 (2.2) | 29.4 (3.3) | | 40.4 (1.7) | 39.6 (3.4) | 42.2 (4.6) |  |
|  | 30 | 3924 | 42.9 (3.3) | | | 50.5 (1.6) | 47.2 (2.6) | | 53.7 (1.2) | 50.3 (2.4) | 54.0 (3.2) |  |
|  | 34 | 3413 | 55.0 (3.4) | | | 58.4 (1.7) | 55.5 (2.7) | | 64.5 (1.3) | 61.0 (2.5) | 67.3 (3.3) |  |
|  | 42 | 3193 | 63.0 (3.6) | | | 65.9 (1.6) | 67.6 (2.6) | | 71.8 (1.2) | 68.4 (2.5) | 72.7 (3.4) |  |
|  |  |  |  | | |  |  | |  |  |  |  |

|  |  | | |  |  | | | Father’s occupational class (10/11y),  Overweight or obese, % (SE) | | | |  |
| --- | --- | --- | --- | --- | --- | --- | --- | --- | --- | --- | --- | --- |
| Cohort | *Gender, age*  Women | N | I | | | II | III NM | | III M | IV | V |  |
| 1946 NSHD | 20 | 1312 | 6.9 (2.6) | | | 9.6 (2.4) | 7.7 (2.3) | | 10.6 (1.6) | 13.8 (2.5) | 18.3 (5.0) |  |
|  | 26 | 1460 | 8.2 (2.8) | | | 14.8 (2.8) | 12.6 (2.6) | | 17.7 (1.8) | 25.7 (3.1) | 28.7 (5.6) |  |
|  | 36 | 1310 | 17.1 (4.1) | | | 20.0 (3.2) | 15.1 (2.9) | | 30.9 (2.4) | 38.0 (3.5) | 33.3 (6.1) |  |
|  | 43 | 1291 | 29.9 (5.2) | | | 36.2 (4.0) | 32.5 (4.0) | | 42.7 (2.5) | 51.9 (3.7) | 52.4 (6.7) |  |
|  | 53 | 1232 | 49.4 (6.3) | | | 60.4 (4.0) | 52.7 (4.4) | | 63.0 (2.6) | 72.2 (3.4) | 64.2 (6.5) |  |
|  | 60-64 | 948 | 62.5 (6.3) | | | 65.1 (4.1) | 57.9 (4.8) | | 73.1 (2.7) | 79.3 (3.6) | 79.8 (7.1) |  |
|  |  |  |  | | |  |  | |  |  |  |  |
| 1958 NCDS | 23 | 3852 | 4.8 (1.8) | | | 10.1 (1.1) | 9.6 (1.5) | | 19.0 (1.0) | 18.4 (1.6) | 16.0 (2.0) |  |
|  | 33 | 3394 | 24.2 (3.7) | | | 28.8 (1.7) | 32.0 (2.5) | | 41.0 (1.3) | 39.0 (2.2) | 39.2 (3.0) |  |
|  | 42 | 3609 | 27.8 (3.7) | | | 34.4 (1.7) | 37.4 (2.5) | | 46.6 (1.3) | 46.9 (2.2) | 42.0 (2.9) |  |
|  | 44 | 3074 | 42.3 (4.2) | | | 50.3 (1.9) | 53.2 (2.7) | | 59.7 (1.4) | 62.9 (2.3) | 56.4 (3.3) |  |
|  | 50 | 2727 | 42.5 (4.4) | | | 50.4 (2.1) | 54.7 (2.9) | | 59.9 (1.5) | 63.4 (2.4) | 55.0 (3.6) |  |
|  |  |  |  | | |  |  | |  |  |  |  |
| 1970 BCS | 26 | 3391 | 13.0 (2.4) | | | 22.0 (1.4) | 19.3 (2.1) | | 28.1 (1.2) | 27.2 (2.3) | 28.1 (3.5) |  |
|  | 30 | 3993 | 24.5 (3.0) | | | 28.8 (1.4) | 28.1 (2.3) | | 37.6 (1.2) | 39.6 (2.3) | 41.4 (3.3) |  |
|  | 34 | 3559 | 32.5 (3.3) | | | 35.6 (1.6) | 36.6 (2.6) | | 45.0 (1.3) | 47.6 (2.6) | 54.7 (3.6) |  |
|  | 42 | 3254 | 44.4 (3.7) | | | 44.4 (1.7) | 40.3 (2.7) | | 54.4 (1.4) | 55.8 (2.6) | 60.1 (3.7) |  |
|  |  |  |  | | |  |  | |  |  |  |  |
