# Supplementary material for: Socioeconomic Inequalities in Body Mass Index across Adulthood: Coordinated Analyses of Individual Participant Data from Three British Birth Cohort Studies Initiated in 1946, 1958 and 1970
Source: PLoS Med. 2017 Jan 10;14(1):e1002214. doi: 10.1371/journal.pmed.1002214 (PMC5224787; doi:10.1371/journal.pmed.1002214)
Supplement: S4 Table — (DOC) [file pmed.1002214.s004.doc]

S4 Table. Own occupational class (42/43y) and overweight or obesity prevalence across adulthood in the 1946 NSHD, 1958 NCDS, and 1970 BCS British birth cohort studies

|  |  | | Own occupational class (42/43y),  Overweight or obese, % (SE) | | | | | | |
| --- | --- | --- | --- | --- | --- | --- | --- | --- | --- |
| *Cohort* | *Gender, age* |  | |  |  |  |  |  |  |
|  | Men | N | | I | II | III NM | III M | IV | V |
| 1946 NSHD | 43 | 1511 | | 52.9 (4.8) | 59.5 (2.4) | 60.2 (5.0) | 62.6 (2.7) | 55.3 (5.3) | 54.0 (9.8) |
|  | 53 | 1281 | | 78.6 (3.8) | 72.5 (2.3) | 74.4 (4.7) | 75.5 (2.6) | 73.1 (5.2) | 72.2 (11.1) |
|  | 60-64 | 961 | | 79.6 (4.1) | 73.5 (2.6) | 76.7 (5.5) | 82.1 (2.7) | 82.0 (5.7) | 63.0 (16.9) |
|  |  |  | |  |  |  |  |  |  |
| 1958 NCDS | 42 | 4607 | | 54.1 (2.7) | 62.3 (1.1) | 60.9 (2.3) | 64.7 (1.3) | 67.3 (2.4) | 62.1 (4.5) |
|  | 44 | 3800 | | 72.4 (2.6) | 76.3 (1.1) | 74.6 (2.3) | 76.6 (1.2) | 77.0 (2.4) | 74.2 (4.6) |
|  | 50 | 3277 | | 68.8 (2.8) | 75.0 (1.2) | 75.2 (2.4) | 78.0 (1.3) | 78.7 (2.6) | 73.2 (5.3) |
|  |  |  | |  |  |  |  |  |  |
| 1970 BCS | 42 | 3627 | | 61.5 (3.0) | 68.4 (1.2) | 68.8 (2.5) | 74.3 (1.4) | 68.4 (2.7) | 64.1 (5.5) |
|  |  |  | |  |  |  |  |  |  |
|  | Women |  | |  |  |  |  |  |  |
| 1946 NSHD | 43 | 1396 | | 48.5 (14.6) | 34.8 (2.7) | 37.0 (2.5) | 43.0 (5.9) | 54.3 (3.9) | 53.7 (6.5) |
|  | 53 | 1260 | | 51.5 (14.7) | 59.5 (2.9) | 63.7 (2.6) | 52.8 (6.4) | 70.3 (3.8) | 75.3 (6.1) |
|  | 60-64 | 993 | | 67.9 (14.0) | 65.2 (3.0) | 71.4 (2.8) | 76.5 (6.1) | 71.0 (4.6) | 82.6 (6.8) |
|  |  |  | |  |  |  |  |  |  |
| 1958 NCDS | 42 | 4075 | | 35.1 (4.6) | 40.2 (1.3) | 41.0 (1.3) | 45.6 (2.9) | 44.9 (1.9) | 55.4 (3.9) |
|  | 44 | 3397 | | 52.8 (4.9) | 55.8 (1.4) | 55.2 (1.5) | 58.0 (3.2) | 59.3 (2.1) | 64.8 (4.2) |
|  | 50 | 2926 | | 52.0 (5.1) | 54.9 (1.5) | 56.8 (1.6) | 58.3 (3.5) | 59.9 (2.3) | 73.1 (4.6) |
|  |  |  | |  |  |  |  |  |  |
| 1970 BCS | 42 | 3337 | | 33.1 (3.7) | 45.9 (1.3) | 52.8 (1.7) | 57.0 (3.4) | 53.8 (2.2) | 60.0 (6.4) |
