# Supplementary material for: Socioeconomic Inequalities in Body Mass Index across Adulthood: Coordinated Analyses of Individual Participant Data from Three British Birth Cohort Studies Initiated in 1946, 1958 and 1970
Source: PLoS Med. 2017 Jan 10;14(1):e1002214. doi: 10.1371/journal.pmed.1002214 (PMC5224787; doi:10.1371/journal.pmed.1002214)
Supplement: S5 Table — (DOC) [file pmed.1002214.s005.doc]

S5 Table. Father’s occupational class (10/11y) and obesity or overweight across adulthood in the 1946 NSHD, 1958 NCDS, and 1970 BCS British birth cohort studies

|  |  |  | Absolute differences in obesity or overweight prevalence in % (95% CI) | | | |  |  |  |
| --- | --- | --- | --- | --- | --- | --- | --- | --- | --- |
| Cohort  1946 NSHD | *Gender, age*  Men | N | I (ref) | II | III NM | III M | IV | V |  |
|  | 20 | 1523 | - | 3.8 (-3.0, 10.6) | 4.6 (-2.6, 11.8) | 7.0 (0.9, 13.1) | 7.8 (0.5, 15.2) | 15.6 (4.8, 26.4) |  |
|  | 26 | 1520 | - | 8.3 (-0.4, 17.0) | 7.3 (-2.0, 16.5) | 16.7 (8.9, 24.4) | 17.0 (7.8, 26.1) | 16.5 (4.8, 28.3) |  |
|  | 36 | 1346 | - | 13.3 (-0.3, 26.9) | 7.9 (-6.0, 21.7) | 23.7 (11.2, 36.3) | 26.8 (13.2, 40.5) | 18.1 (1.7, 34.5) |  |
|  | 43 | 1328 | - | 14.2 (-0.4, 28.7) | 9.4 (-5.5, 24.2) | 23.9 (10.5, 37.4) | 19.3 (4.7, 33.8) | 16.4 (-0.8, 33.5) |  |
|  | 53 | 1202 | - | 1.5 (-12.4, 15.5) | -1.5 (-16.0, 13.0) | 12.7 (-0.2, 25.5) | 10.7 (-2.9, 24.4) | 5.7 (-10.3, 21.7) |  |
|  | 60-64 | 896 | - | 3.0 (-11.7, 17.8) | -2.8 (-18.3, 12.8) | 16.5 (3.1, 29.9) | 9.7 (-5.0, 24.4) | -9.0 (-28.1, 10.2) |  |
|  |  |  |  |  |  |  |  |  |  |
| 1958 NCDS | 23 | 3954 | - | 6.1 (1.0, 11.3) | 5.0 (-0.7, 10.6) | 11.8 (6.9, 16.7) | 10.4 (4.7, 16.0) | 12.2 (5.9, 18.4) |  |
|  | 33 | 3508 | - | 3.6 (-4.6, 11.8) | 6.5 (-2.4, 15.5) | 8.7 (0.9, 16.5) | 11.8 (3.2, 20.4) | 13.8 (4.5, 23.1) |  |
|  | 42 | 3622 | - | 3.7 (-4.4, 11.7) | 5.5 (-3.2, 14.2) | 5.5 (-2.2, 13.2) | 6.3 (-2.1, 14.8) | 12.9 (4.0, 21.8) |  |
|  | 44 | 3039 | - | 3.0 (-5.1, 11.2) | 5.1 (-3.5, 13.8) | 6.5 (-1.2, 14.2) | 10.6 (2.2, 18.9) | 9.5 (0.6, 18.4) |  |
|  | 50 | 2758 | - | 1.4 (-7.2, 9.9) | 2.6 (-6.6, 11.8) | 7.0 (-1.1, 15.0) | 9.8 (1.1, 18.6) | 11.9 (2.7, 21.1) |  |
|  |  |  |  |  |  |  |  |  |  |
| 1970 BCS | 26 | 1901 | - | 6.2 (-3.0, 15.4) | 2.1 (-8.2, 12.4) | 13.1 (4.3, 21.8) | 12.3 (1.8, 22.7) | 14.9 (2.8, 27.0) |  |
|  | 30 | 3924 | - | 7.6 (0.4, 14.7) | 4.2 (-4.0, 12.5) | 10.8 (3.9, 17.7) | 7.4 (-0.5, 15.4) | 11.1 (2.1, 20.0) |  |
|  | 34 | 3413 | - | 3.4 (-4.0, 10.9) | 0.5 (-8.1, 9.0) | 9.5 (2.3, 16.7) | 6.0 (-2.4, 14.3) | 12.4 (3.0, 21.7) |  |
|  | 42 | 3193 | - | 2.9 (-4.8, 10.6) | 4.6 (-4.1, 13.3) | 8.9 (1.4, 16.3) | 5.4 (-3.2, 13.9) | 9.7 (0.1, 19.4) |  |

|  |  |  | Absolute differences in obesity or overweight prevalence in % (95% CI) | | | | |  |  |  |
| --- | --- | --- | --- | --- | --- | --- | --- | --- | --- | --- |
| Cohort  1946 NSHD | *Gender, age*  Women | N | I (ref) |  | II | III NM | III M | IV | V |  |
|  | 20 | 1312 | - |  | 2.7 (-4.2, 9.6) | 0.8 (-5.9, 7.5) | 3.7 (-2.2, 9.6) | 6.8 (-0.2, 13.9) | 11.3 (0.2, 22.4) |  |
|  | 26 | 1460 | - |  | 6.6 (-1.2, 14.4) | 4.4 (-3.0, 11.9) | 9.5 (3.0, 16.1) | 17.5 (9.3, 25.8) | 20.6 (8.4, 32.8) |  |
|  | 36 | 1310 | - |  | 3.0 (-7.3, 13.2) | -1.9 (-11.8, 8.0) | 13.8 (4.5, 23.1) | 21.0 (10.3, 31.7) | 16.3 (1.8, 30.8) |  |
|  | 43 | 1291 | - |  | 6.3 (-6.6, 19.2) | 2.6 (-10.3, 15.5) | 12.8 (1.4, 24.1) | 22.0 (9.5, 34.5) | 22.5 (5.9, 39.2) |  |
|  | 53 | 1232 | - |  | 10.9 (-3.6, 25.5) | 3.3 (-11.8, 18.4) | 13.5 (0.2, 26.9) | 22.8 (8.8, 36.8) | 14.8 (-3.0, 32.5) |  |
|  | 60-64 | 948 | - |  | 2.6 (-12.1, 17.3) | -4.6 (-20.2, 10.9) | 10.6 (-2.9, 24.0) | 16.8 (2.6, 30.9) | 17.3 (-1.2, 35.9) |  |
|  |  |  |  |  |  |  |  |  |  |  |
| 1958 NCDS | 23 | 3852 | - |  | 5.4 (1.3, 9.4) | 4.9 (0.4, 9.3) | 14.3 (10.3, 18.2) | 13.6 (8.9, 18.3) | 11.2 (5.9, 16.5) |  |
|  | 33 | 3394 | - |  | 4.6 (-3.5, 12.6) | 7.8 (-1.0, 16.6) | 16.8 (9.0, 24.5) | 14.7 (6.2, 23.2) | 15.0 (5.6, 24.3) |  |
|  | 42 | 3609 | - |  | 6.6 (-1.3, 14.5) | 9.6 (1.0, 18.2) | 18.7 (11.2, 26.3) | 19.1 (10.8, 27.5) | 14.2 (5.1, 23.3) |  |
|  | 44 | 3074 | - |  | 8.0 (-1.2, 17.1) | 10.9 (1.1, 20.7) | 17.4 (8.7, 26.1) | 20.6 (11.1, 30.0) | 14.1 (3.6, 24.6) |  |
|  | 50 | 2727 | - |  | 7.9 (-1.6, 17.4) | 12.2 (1.9, 22.5) | 17.4 (8.3, 26.4) | 20.8 (11.0, 30.7) | 12.5 (1.3, 23.6) |  |
|  |  |  |  |  |  |  |  |  |  |  |
| 1970 BCS | 26 | 3391 | - |  | 9.0 (3.5, 14.5) | 6.4 (0.1, 12.6) | 15.2 (9.9, 20.5) | 14.2 (7.7, 20.8) | 15.2 (6.9, 23.5) |  |
|  | 30 | 3993 | - |  | 4.3 (-2.2, 10.7) | 3.6 (-3.8, 10.9) | 13.0 (6.8, 19.3) | 15.0 (7.7, 22.4) | 16.8 (8.1, 25.6) |  |
|  | 34 | 3559 | - |  | 3.1 (-4.0, 10.2) | 4.1 (-4.1, 12.2) | 12.5 (5.6, 19.4) | 15.1 (7.0, 23.3) | 22.2 (12.6, 31.8) |  |
|  | 42 | 3254 | - |  | -0.1 (-8.1, 7.9) | -4.2 (-13.2, 4.8) | 10.0 (2.2, 17.7) | 11.3 (2.5, 20.2) | 15.7 (5.4, 25.9) |  |

Note: Absolute differences estimated using linear probability models; relative differences estimated using generalized linear models.
