# Supplementary material for: Socioeconomic Inequalities in Body Mass Index across Adulthood: Coordinated Analyses of Individual Participant Data from Three British Birth Cohort Studies Initiated in 1946, 1958 and 1970
Source: PLoS Med. 2017 Jan 10;14(1):e1002214. doi: 10.1371/journal.pmed.1002214 (PMC5224787; doi:10.1371/journal.pmed.1002214)
Supplement: S6 Table — (DOC) [file pmed.1002214.s006.doc]

S6 Table. Father’s occupational class (10/11y) and BMI across adulthood (≥20 years) in the 1946 NSHD, 1958 NCDS, and 1970 BCS British birth cohort studies: estimates from separate multilevel models, scaled to show estimated BMI differences at 26 years

|  |  | Cohort |  |  |
| --- | --- | --- | --- | --- |
|  | **Men** | 1946 NSHD | 1958 NCDS | 1970 BCS |
| N participants, observations |  | 1,901, 7,921 | 4,802, 17,042 | 4,732, 12,526 |
| Main effect: age |  | .14*** (0.01) | .22*** (0.01) | .3*** (0.017) |
| Main effect: age^2^ |  | -.00042** (0.00014) | -.0024*** (0.00018) | -.0086*** (0.0005) |
| Main effect: SEP | Class I (ref) |  |  |  |
|  | II | .65* (0.26) | 0.37 (0.23) | .59* (0.25) |
|  | III NM | 0.36 (0.28) | 0.43 (0.25) | 0.22 (0.29) |
|  | III M | 1*** (0.25) | .85*** (0.22) | .82*** (0.24) |
|  | IV | 1.2*** (0.27) | .79*** (0.24) | .79** (0.28) |
|  | V | 1.3*** (0.33) | .87*** (0.25) | .92** (0.32) |
|  |  |  |  |  |
| SEP*age interactions | II | -0.0088 (0.011) | .023* (0.011) | -0.0039 (0.016) |
|  | III NM | -0.0014 (0.011) | .025* (0.012) | 0.022 (0.018) |
|  | III M | 0.012 (0.01) | .025* (0.01) | .033* (0.016) |
|  | IV | 0.011 (0.011) | .033** (0.011) | .04* (0.018) |
|  | V | -0.015 (0.014) | .031* (0.012) | 0.024 (0.021) |
|  |  |  |  |  |
| Constant |  | 23*** (0.23) | 23*** (0.21) | 24*** (0.23) |
| Random effects | sd(xage) | .097*** (0.0027) | .1*** (0.0021) | .16*** (0.0037) |
|  | sd(_cons) | 2.5*** (0.058) | 2.7*** (0.036) | 3.4*** (0.043) |
|  | sd(Residual) | 1.1*** (0.023) | 1.6*** (0.012) | 1.3*** (0.014) |
|  |  |  |  |  |

|  |  | Cohort |  |  |
| --- | --- | --- | --- | --- |
|  | **Women** | 1946 NSHD | 1958 NCDS | 1970 BCS |
| N participants, observations |  | 1,782, 7,830 | 4,694, 17,165 | 4,899, 14,631 |
| Main effect: age |  | .11*** (0.013) | .19*** (0.014) | .26*** (0.018) |
| Main effect: age^2^ |  | .00084*** (0.00017) | -.0017*** (0.00021) | -.0056*** (0.00051) |
| Main effect: SEP | Class I (ref) |  |  |  |
|  | II | 0.2 (0.28) | .6* (0.29) | .6* (0.29) |
|  | III NM | 0.024 (0.27) | .63* (0.31) | 0.55 (0.33) |
|  | III M | .67* (0.27) | 1.5*** (0.27) | 1.2*** (0.28) |
|  | IV | 1.2*** (0.29) | 1.4*** (0.3) | 1.3*** (0.32) |
|  | V | 1.5*** (0.43) | 1.1*** (0.32) | 1.8*** (0.37) |
|  |  |  |  |  |
| SEP*age interactions | II | 0.0063 (0.014) | 0.012 (0.014) | 0.0011 (0.018) |
|  | III NM | 0.011 (0.014) | 0.027 (0.015) | -0.0072 (0.02) |
|  | III M | .045** (0.014) | .043** (0.014) | .04* (0.017) |
|  | IV | .053*** (0.015) | .051*** (0.015) | 0.022 (0.02) |
|  | V | .05* (0.021) | 0.014 (0.016) | .062** (0.023) |
|  |  |  |  |  |
| Constant |  | 22*** (0.23) | 22*** (0.26) | 22*** (0.27) |
| Random effects | sd(xage) | .14*** (0.0049) | .13*** (0.0026) | .18*** (0.004) |
|  | sd(_cons) | 3*** (0.094) | 3.2*** (0.043) | 4*** (0.048) |
|  | sd(Residual) | 1.5*** (0.03) | 1.9*** (0.015) | 1.6*** (0.015) |

Estimates and standard errors shown in parentheses; *p<0.05,**p<0.01,***p<0.001.
