# Supplementary material for: Socioeconomic Inequalities in Body Mass Index across Adulthood: Coordinated Analyses of Individual Participant Data from Three British Birth Cohort Studies Initiated in 1946, 1958 and 1970
Source: PLoS Med. 2017 Jan 10;14(1):e1002214. doi: 10.1371/journal.pmed.1002214 (PMC5224787; doi:10.1371/journal.pmed.1002214)
Supplement: S7 Table — (DOC) [file pmed.1002214.s007.doc]

S7 Table. Father’s occupational class (10/11y) and BMI across adulthood (≥20 years) in the 1946 NSHD, 1958 NCDS, and 1970 BCS British birth cohort studies, adjusted for adult occupational class (42/43y): estimates from separate multilevel models, scaled to show estimated BMI differences at 26 years

|  | Cohort |  | |  | |  | |  | |  | |  | |
| --- | --- | --- | --- | --- | --- | --- | --- | --- | --- | --- | --- | --- | --- |
| **Men** | 1946 NSHD | |  | | 1958 NCDS | |  | | 1970 BCS | |  | |  |
| N participants, observations | 1,280, 6588 | | 1,280, 6588 | | 3,364, 14053 | | 3,364, 14053 | | 3,129, 9465 | | 3,129, 9465 | |  |
| Main effect: age | .14*** (0.0098) | | .14*** (0.0098) | | .22*** (0.011) | | .22*** (0.011) | | .22*** (0.011) | | .29*** (0.018) | |  |
| Main effect: age^2^ | -.00047** (0.00015) | | -.00047** (0.00015) | | -.0023*** (0.00018) | | -.0023*** (0.00018) | | -.0023*** (0.00018) | | -.0085*** (0.00054) | |  |
| Main effect: childhood SEP  (class I ref) |  | |  | |  | |  | |  | |  | |  |
| II | 0.37 (0.3) | | 0.31 (0.3) | | 0.28 (0.25) | | 0.21 (0.25) | | 0.51 (0.29) | | 0.47 (0.29) | |  |
| III NM | 0.2 (0.32) | | 0.13 (0.32) | | 0.17 (0.27) | | 0.062 (0.27) | | 0.41 (0.33) | | 0.34 (0.33) | |  |
| III M | .94** (0.3) | | .81** (0.31) | | .61* (0.24) | | 0.4 (0.24) | | .84** (0.28) | | .73* (0.29) | |  |
| IV | 1.2*** (0.33) | | 1** (0.34) | | .71** (0.26) | | .52* (0.26) | | 0.58 (0.33) | | 0.48 (0.34) | |  |
| V | 1.3*** (0.39) | | 1.1** (0.41) | | .73* (0.29) | | 0.52 (0.29) | | 0.65 (0.39) | | 0.57 (0.39) | |  |
| Childhood SEP*age interactions |  | |  | |  | |  | |  | |  | |  |
| II | -0.00038 (0.011) | | -0.00033 (0.011) | | 0.019 (0.011) | | 0.02 (0.011) | | 0.02 (0.011) | | 0.0026 (0.016) | |  |
| III NM | 0.0068 (0.011) | | 0.0068 (0.011) | | 0.022 (0.012) | | 0.022 (0.012) | | 0.022 (0.012) | | 0.036 (0.018) | |  |
| III M | .023* (0.01) | | .023* (0.01) | | .023* (0.011) | | .023* (0.011) | | .023* (0.011) | | .04* (0.016) | |  |
| IV | 0.015 (0.011) | | 0.015 (0.011) | | .034** (0.012) | | .034** (0.012) | | .034** (0.012) | | .039* (0.018) | |  |
| V | -0.0073 (0.013) | | -0.0075 (0.013) | | .032* (0.013) | | .032* (0.013) | | .032* (0.013) | | 0.035 (0.021) | |  |
| Main effect: adult SEP |  | |  | |  | |  | |  | |  | |  |
| II |  | | 0.25 (0.22) | |  | | 0.33 (0.2) | |  | | 0.38 (0.25) | |  |
| III NM |  | | 0.22 (0.29) | |  | | 0.36 (0.24) | |  | | .75* (0.31) | |  |
| III M |  | | .49* (0.25) | |  | | .77*** (0.21) | |  | | .79** (0.27) | |  |
| IV |  | | 0.025 (0.34) | |  | | .92*** (0.26) | |  | | 0.34 (0.33) | |  |
| V |  | | 1.1 (0.75) | |  | | 0.25 (0.37) | |  | | -0.61 (0.52) | |  |
|  |  | |  | |  | |  | |  | |  | |  |
| Constant | 23*** (0.27) | | 22*** (0.3) | | 23*** (0.22) | | 23*** (0.27) | | 24*** (0.26) | | 24*** (0.33) | |  |
| sd(xage) | .092*** (0.0027) | | .092*** (0.0027) | | .099*** (0.0022) | | .099*** (0.0022) | | .14*** (0.0037) | | .14*** (0.0037) | |  |
| sd(_cons) | 2.5*** (0.062) | | 2.5*** (0.061) | | 2.6*** (0.04) | | 2.6*** (0.04) | | 3.4*** (0.052) | | 3.4*** (0.052) | |  |
| sd(Residual) | 1.2*** (0.024) | | 1.2*** (0.024) | | 1.5*** (0.012) | | 1.5*** (0.012) | | 1.3*** (0.016) | | 1.3*** (0.016) | |  |
|  |  |  | |  | |  | |  | |  | |  | |

|  | Cohort |  | |  | |  | |  | |  | |  | |
| --- | --- | --- | --- | --- | --- | --- | --- | --- | --- | --- | --- | --- | --- |
| **Women** | 1946 NSHD | |  | | 1958 NCDS | |  | | 1970 BCS | |  | |  |
| N participants, observations | 1199, 6242 | | 1199, 6242 | | 3050, 12,835 | | 3050, 12,835 | | 3,025, 10,168 | | 3,025, 10,168 | |  |
| Main effect: age | .11*** (0.015) | | .11*** (0.015) | | .19*** (0.015) | | .19*** (0.015) | | .19*** (0.015) | | .26*** (0.02) | |  |
| Main effect: age^2^ | .00097*** (0.00018) | | .00097*** (0.00018) | | -.0015*** (0.00024) | | -.0015*** (0.00024) | | -.0015*** (0.00024) | | -.0056*** (0.00057) | |  |
| Main effect: childhood SEP  (class I ref) |  | |  | |  | |  | |  | |  | |  |
| II | 0.24 (0.31) | | 0.13 (0.31) | | 0.62 (0.33) | | 0.59 (0.33) | | 0.4 (0.35) | | 0.2 (0.35) | |  |
| III NM | -0.021 (0.3) | | -0.15 (0.31) | | 0.59 (0.36) | | 0.54 (0.36) | | 0.34 (0.4) | | 0.092 (0.4) | |  |
| III M | .61* (0.29) | | 0.31 (0.29) | | 1.5*** (0.32) | | 1.3*** (0.32) | | .91** (0.34) | | 0.57 (0.34) | |  |
| IV | 1.4*** (0.34) | | 1** (0.33) | | 1.4*** (0.34) | | 1.3*** (0.35) | | 1.1** (0.39) | | 0.75 (0.39) | |  |
| V | 1.1* (0.52) | | 0.54 (0.54) | | 1** (0.38) | | .83* (0.38) | | 1.4** (0.47) | | 1* (0.47) | |  |
| Childhood SEP*age interactions |  | |  | |  | |  | |  | |  | |  |
| II | -0.0008 (0.016) | | -0.00073 (0.016) | | 0.017 (0.016) | | 0.017 (0.016) | | 0.017 (0.016) | | 0.0076 (0.019) | |  |
| III NM | -0.0036 (0.016) | | -0.0036 (0.016) | | 0.026 (0.017) | | 0.026 (0.017) | | 0.026 (0.017) | | -0.0082 (0.022) | |  |
| III M | .035* (0.016) | | .035* (0.016) | | .043** (0.015) | | .043** (0.015) | | .043** (0.015) | | .037* (0.019) | |  |
| IV | .04* (0.017) | | .04* (0.017) | | .054*** (0.016) | | .054*** (0.016) | | .054*** (0.016) | | 0.022 (0.022) | |  |
| V | 0.02 (0.022) | | 0.02 (0.022) | | 0.0063 (0.018) | | 0.0056 (0.018) | | 0.0056 (0.018) | | .052* (0.026) | |  |
| Main effect: adult SEP |  | |  | |  | |  | |  | |  | |  |
| II |  | | -0.3 (0.49) | |  | | 0.14 (0.37) | |  | | 1.2*** (0.35) | |  |
| III NM |  | | -0.2 (0.49) | |  | | 0.13 (0.37) | |  | | 1.6*** (0.36) | |  |
| III M |  | | 0.67 (0.58) | |  | | 0.62 (0.42) | |  | | 1.8*** (0.45) | |  |
| IV |  | | 0.82 (0.54) | |  | | 0.6 (0.39) | |  | | 1.9*** (0.39) | |  |
| V |  | | 0.9 (0.6) | |  | | 1.5** (0.47) | |  | | 2.7*** (0.67) | |  |
|  |  | |  | |  | |  | |  | |  | |  |
| Constant | 22*** (0.25) | | 22*** (0.53) | | 22*** (0.3) | | 21*** (0.44) | | 23*** (0.32) | | 22*** (0.42) | |  |
| sd(xage) | .13*** (0.0046) | | .13*** (0.0046) | | .12*** (0.0028) | | .12*** (0.0028) | | .17*** (0.0042) | | .17*** (0.0042) | |  |
| sd(_cons) | 2.8*** (0.11) | | 2.8*** (0.1) | | 3.1*** (0.051) | | 3*** (0.05) | | 3.9*** (0.059) | | 3.9*** (0.059) | |  |
| sd(Residual) | 1.5*** (0.032) | | 1.5*** (0.032) | | 1.9*** (0.016) | | 1.9*** (0.016) | | 1.6*** (0.017) | | 1.6*** (0.017) | |  |
|  |  |  | |  | |  | |  | |  | |  | |

Estimates and standard errors shown in parentheses; *p<0.05,**p<0.01,***p<0.001.
