# Supplementary material for: Socioeconomic Inequalities in Body Mass Index across Adulthood: Coordinated Analyses of Individual Participant Data from Three British Birth Cohort Studies Initiated in 1946, 1958 and 1970
Source: PLoS Med. 2017 Jan 10;14(1):e1002214. doi: 10.1371/journal.pmed.1002214 (PMC5224787; doi:10.1371/journal.pmed.1002214)
Supplement: S8 Table — (DOC) [file pmed.1002214.s008.doc]

S8 Table. Own occupational class (42/43y) and obesity or overweight across mid-adulthood in the 1946 NSHD, 1958 NCDS, and 1970 BCS birth cohort studies

|  |  |  | Absolute differences in obesity or overweight prevalence in % (95% CI) | | | |  |  |
| --- | --- | --- | --- | --- | --- | --- | --- | --- |
| Cohort | *Gender, age*  Men | N | I (ref) | II | III NM | III M | IV | V |
| 1946 NSHD | 43 | 1511 | - | 6.5 (-3.9, 17.0) | 7.3 (-6.3, 20.9) | 9.6 (-1.1, 20.3) | 2.4 (-11.6, 16.3) | 1.1 (-20.3, 22.5) |
|  | 53 | 1281 | - | -6.1 (-14.9, 2.6) | -4.2 (-16.1, 7.8) | -3.1 (-12.2, 5.9) | -5.5 (-18.2, 7.2) | -6.4 (-29.5, 16.7) |
|  | 60-64 | 961 | - | -6.1 (-15.6, 3.4) | -2.9 (-16.3, 10.6) | 2.5 (-7.1, 12.1) | 2.4 (-11.3, 16.1) | -16.6 (-50.8, 17.6) |
|  |  |  |  |  |  |  |  |  |
| 1958 NCDS | 42 | 4607 | - | 8.2 (2.4, 13.9) | 6.7 (-0.2, 13.7) | 10.6 (4.7, 16.4) | 13.1 (6.0, 20.2) | 7.9 (-2.4, 18.2) |
|  | 44 | 3800 | - | 3.9 (-1.6, 9.4) | 2.2 (-4.5, 8.9) | 4.2 (-1.5, 9.8) | 4.6 (-2.4, 11.5) | 1.8 (-8.5, 12.1) |
|  | 50 | 3277 | - | 6.2 (0.2, 12.2) | 6.4 (-0.9, 13.6) | 9.2 (3.1, 15.3) | 9.9 (2.4, 17.4) | 4.5 (-7.2, 16.2) |
|  |  |  |  |  |  |  |  |  |
| 1970 BCS | 42 | 3627 | - | 6.9 (0.6, 13.2) | 7.3 (-0.4, 14.9) | 12.8 (6.3, 19.3) | 6.9 (-1.0, 14.8) | 2.6 (-9.6, 14.8) |
|  |  |  |  |  |  |  |  |  |
|  | Women |  |  |  |  |  |  |  |
| 1946 NSHD | 43 | 1396 | - | -13.7 (-42.8, 15.4) | -11.5 (-40.6, 17.5) | -5.4 (-36.4, 25.5) | 5.8 (-23.8, 35.4) | 5.2 (-26.2, 36.5) |
|  | 53 | 1260 | - | 8.0 (-21.4, 37.4) | 12.2 (-17.1, 41.5) | 1.3 (-30.2, 32.7) | 18.7 (-11.1, 48.5) | 23.8 (-7.5, 55.0) |
|  | 60-64 | 993 | - | -2.6 (-30.8, 25.5) | 3.5 (-24.6, 31.6) | 8.6 (-21.4, 38.7) | 3.2 (-25.8, 32.1) | 14.8 (-15.8, 45.4) |
|  |  |  |  |  |  |  |  |  |
| 1958 NCDS | 42 | 4075 | - | 5.0 (-4.2, 14.3) | 5.8 (-3.4, 15.1) | 10.4 (-0.2, 21.0) | 9.8 (0.2, 19.4) | 20.2 (8.6, 31.9) |
|  | 44 | 3397 | - | 3.0 (-6.9, 12.9) | 2.4 (-7.5, 12.3) | 5.2 (-6.2, 16.6) | 6.4 (-3.9, 16.8) | 12.0 (-0.6, 24.6) |
|  | 50 | 2926 | - | 2.8 (-7.5, 13.2) | 4.8 (-5.6, 15.2) | 6.3 (-5.7, 18.3) | 7.8 (-3.0, 18.7) | 21.1 (7.7, 34.5) |
|  |  |  |  |  |  |  |  |  |
| 1970 BCS | 42 | 3337 | - | 12.8 (5.1, 20.4) | 19.7 (11.7, 27.6) | 23.9 (14.1, 33.7) | 20.7 (12.2, 29.1) | 26.9 (12.5, 41.2) |

Note: Absolute differences estimated using linear probability models; relative differences estimated using generalized linear models.
