# Supplementary material for: Socioeconomic Inequalities in Body Mass Index across Adulthood: Coordinated Analyses of Individual Participant Data from Three British Birth Cohort Studies Initiated in 1946, 1958 and 1970
Source: PLoS Med. 2017 Jan 10;14(1):e1002214. doi: 10.1371/journal.pmed.1002214 (PMC5224787; doi:10.1371/journal.pmed.1002214)
Supplement: S9 Table — (DOC) [file pmed.1002214.s009.doc]

S9 Table. Own occupational class (42/43 years) and adult BMI (≥42 years) in the 1946 NSHD and 1958 NCDS British birth cohort studies: estimates from separate multilevel models, scaled to show estimated BMI differences at 43 years

|  |  | Cohort |  |  |
| --- | --- | --- | --- | --- |
|  | **Men** | 1946 NSHD | 1958 NCDS | 1970 BCS |
| N participants, observations |  | 1,532, 3,766 | 4,706, 11,714 | 3636 |
| Main effect: age |  | .14*** (0.0099) | .14*** (0.016) |  |
| Main effect: age^2^ |  | 25*** (0.23) | 26*** (0.21) |  |
| Main effect: SEP | Class I (ref) |  |  |  |
|  | II | .73** (0.26) | .72** (0.23) | .82** (0.3) |
|  | III NM | .79* (0.39) | .82** (0.27) | 1** (0.36) |
|  | III M | 1*** (0.28) | .87*** (0.23) | 1.4*** (0.31) |
|  | IV | 0.69 (0.42) | .98*** (0.28) | 1** (0.38) |
|  | V | 0.95 (0.79) | 0.55 (0.41) | 0.39 (0.57) |
|  |  |  |  |  |
| SEP*age interactions | II | -0.0099 (0.012) | 0.023 (0.017) |  |
|  | III NM | -0.019 (0.017) | 0.011 (0.021) |  |
|  | III M | -0.00078 (0.014) | .066*** (0.018) |  |
|  | IV | -0.003 (0.02) | .084*** (0.022) |  |
|  | V | 0.0051 (0.048) | .096** (0.034) |  |
|  |  |  |  |  |
| Constant |  | 25*** (0.23) | 26*** (0.21) | 27*** (0.28) |
| Random effects | sd(xage) | .15*** (0.0053) | .092*** (0.012) |  |
|  | sd(_cons) | 3.4*** (0.084) | 3.7*** (0.042) |  |
|  | sd(Residual) | .83*** (0.032) | 1.5*** (0.018) |  |
|  |  |  |  |  |

|  |  | Cohort |  |  |
| --- | --- | --- | --- | --- |
|  | **Women** | 1946 NSHD, | 1958 NCDS | 1970 BCS |
| N participants, observations |  | 1,439, 3,682 | 4,245, 10,518 | 3,386 |
| Main effect: age |  | .12* (0.047) | .13*** (0.031) |  |
| Main effect: age^2^ |  | 26*** (0.86) | 24*** (0.44) |  |
| Main effect: SEP | Class I (ref) |  |  |  |
|  | II | -1.3 (0.88) | .99* (0.46) | 1.5*** (0.43) |
|  | III NM | -1 (0.88) | 0.79 (0.46) | 2.1*** (0.45) |
|  | III M | 0.043 (1) | 1.8*** (0.53) | 2.8*** (0.54) |
|  | IV | 0.68 (0.94) | 1.3** (0.48) | 2.6*** (0.47) |
|  | V | 1.2 (1.1) | 2.4*** (0.58) | 3.9*** (0.8) |
|  |  |  |  |  |
| SEP*age interactions | II | 0.052 (0.048) | 0.034 (0.032) |  |
|  | III NM | 0.062 (0.048) | 0.05 (0.032) |  |
|  | III M | 0.043 (0.052) | 0.049 (0.037) |  |
|  | IV | 0.059 (0.05) | 0.056 (0.034) |  |
|  | V | 0.063 (0.056) | 0.037 (0.044) |  |
|  |  |  |  |  |
| Constant |  | 26*** (0.86) | 24*** (0.44) | 24*** (0.41) |
| Random effects | sd(xage) | .19*** (0.0072) | .56*** (0.079) |  |
|  | sd(_cons) | 4.4*** (0.15) | .073*** (0.011) |  |
|  | sd(Residual) | 1.1** (0.04) | 1.6*** (0.1) |  |

Estimates and standard errors shown in parentheses; *p<0.05,**p<0.01,***p<0.001; only fixed effects shown for the 1970 BCS since only one age point of BMI was available
