# Supplementary material for: Socioeconomic Inequalities in Body Mass Index across Adulthood: Coordinated Analyses of Individual Participant Data from Three British Birth Cohort Studies Initiated in 1946, 1958 and 1970
Source: PLoS Med. 2017 Jan 10;14(1):e1002214. doi: 10.1371/journal.pmed.1002214 (PMC5224787; doi:10.1371/journal.pmed.1002214)
Supplement: S10 Table — (DOC) [file pmed.1002214.s010.doc]

S10 Table. Socioeconomic position in relation to calculated change in BMI between a self-reported (at 42 years) and objective measure (at 44 years) in the 1958 NCDS British birth cohort study

|  |  |  | kg/m^2^ BMI difference (95% CI) | | | |  |  |
| --- | --- | --- | --- | --- | --- | --- | --- | --- |
|  |  | N | I (ref) | II | III NM | III M | IV | V |
| Father’s occupational class (10/11y) | Women | 2,938 | - | 0.3 (-0.2, 0.7) | 0.1 (-0.4, 0.6) | 0.3 (-0.1, 0.8) | 0.5 (-0.0, 0.9) | 0.1 (-0.4, 0.6) |
|  | Men | 2,893 |  | 0.0 (-0.4, 0.3) | 0.0 (-0.4, 0.4) | 0.0 (-0.4, 0.4) | 0.0 (-0.4, 0.4) | 0.0 (-0.4, 0.4) |
|  |  |  |  |  |  |  |  |  |
| Own occupational class (42/43y) | Women | 3,321 |  | 0.2 (-0.3, 0.7) | 0.2 (-0.3, 0.7) | 0.3 (-0.3, 0.9) | 0.3 (-0.2, 0.8) | -0.2 (-0.9, 0.4) |
|  | Men | 3,726 | - | 0.1 (-0.2, 0.3) | -0.1 (-0.4, 0.2) | 0.0 (-0.3, 0.2) | 0.2 (-0.1, 0.5) | -0.1 (-0.6, 0.4) |

Note: BMI change score mean (SD) = 1.5 (2.0) in men, and 1.8 (2.5) in women.
