# Supplementary material for: Socioeconomic Inequalities in Body Mass Index across Adulthood: Coordinated Analyses of Individual Participant Data from Three British Birth Cohort Studies Initiated in 1946, 1958 and 1970
Source: PLoS Med. 2017 Jan 10;14(1):e1002214. doi: 10.1371/journal.pmed.1002214 (PMC5224787; doi:10.1371/journal.pmed.1002214)
Supplement: S1 PRIMSA Checklist — (DOC) [file pmed.1002214.s011.doc]

STROBE Statement—Checklist of items that should be included in reports of ***cohort studies***

|  | Item No | Recommendation |  |
| --- | --- | --- | --- |
| **Title and abstract** | 1 | (*a*) Indicate the study’s design with a commonly used term in the title or the abstract | Title page and abstract |
|  |  | (*b*) Provide in the abstract an informative and balanced summary of what was done and what was found | Abstract |
| Introduction | | |  |
| Background/rationale | 2 | Explain the scientific background and rationale for the investigation being reported | Introduction paragraphs 1-3 |
| Objectives | 3 | State specific objectives, including any prespecified hypotheses | Introduction paragraph 3 “we hypothesised…” |
| Methods | | |  |
| Study design | 4 | Present key elements of study design early in the paper | Methods paragraph 1 “Britain’s birth cohort studies followed-up to adulthood were used…” |
| Setting | 5 | Describe the setting, locations, and relevant dates, including periods of recruitment, exposure, follow-up, and data collection | Methods paragraph 1 “Britain’s birth cohort studies followed-up to adulthood were used…” |
| Participants | 6 | (*a*) Give the eligibility criteria, and the sources and methods of selection of participants. Describe methods of follow-up | Methods paragraph 1 “These were designed to be nationally representative when initiated in 1946…” |
|  |  | (*b*) For matched studies, give matching criteria and number of exposed and unexposed |  |
| Variables | 7 | Clearly define all outcomes, exposures, predictors, potential confounders, and effect modifiers. Give diagnostic criteria, if applicable | Methods – BMI and SEP sub-sections. |
| Data sources/ measurement | 8* | For each variable of interest, give sources of data and details of methods of assessment (measurement). Describe comparability of assessment methods if there is more than one group | Methods – BMI and SEP sub-sections. |
| Bias | 9 | Describe any efforts to address potential sources of bias | Methods, sensitivity analyses sub-section “To examine the extent to which self-reported BMI data could bias SEP and BMI associations” |
| Study size | 10 | Explain how the study size was arrived at | Methods paragraph 1 “analytic sample sizes were …” |
| Quantitative variables | 11 | Explain how quantitative variables were handled in the analyses. If applicable, describe which groupings were chosen and why | Methods, analytical strategy section |
| Statistical methods | 12 | (*a*) Describe all statistical methods, including those used to control for confounding | Methods, analytical strategy section |
|  |  | (*b*) Describe any methods used to examine subgroups and interactions | Methods, analytical strategy section |
|  |  | (*c*) Explain how missing data were addressed | Methods, analytical strategy section |
|  |  | (*d*) If applicable, explain how loss to follow-up was addressed | Methods, analytical strategy section |
|  |  | (*e*) Describe any sensitivity analyses | Methods, sensitivity analyses section |
| Results | | |  |
| Participants | 13* | (a) Report numbers of individuals at each stage of study—eg numbers potentially eligible, examined for eligibility, confirmed eligible, included in the study, completing follow-up, and analysed | Methods, paragraph 1 “analyses were restricted to singleton births in England, Scotland and Wales from those born and included in cohorts in the relevant weeks in March/April 1946 (N=5,362), 1958 (N=16,383) and 1970 (N=16,172). The analytic sample sizes were…” |
|  |  | (b) Give reasons for non-participation at each stage | N/A – The number of separate follow-ups is too large to detail this in every instance, for each study—the cohort profiles have been cited which provide this information |
|  |  | (c) Consider use of a flow diagram | - |
| Descriptive data | 14* | (a) Give characteristics of study participants (eg demographic, clinical, social) and information on exposures and potential confounders | Results, paragraph 1 “BMI was typically higher at older ages…” |
|  |  | (b) Indicate number of participants with missing data for each variable of interest | Table 1 |
|  |  | (c) Summarise follow-up time (eg, average and total amount) | Methods, BMI sub-section and Table 1 |
| Outcome data | 15* | Report numbers of outcome events or summary measures over time | Table 1 |
| Main results | 16 | (*a*) Give unadjusted estimates and, if applicable, confounder-adjusted estimates and their precision (eg, 95% confidence interval). Make clear which confounders were adjusted for and why they were included | Table 1, Table 2 |
|  |  | (*b*) Report category boundaries when continuous variables were categorized | Methods, analytical strategy sub-section “binary outcome indicating normal (BMI<25) or overweight-obese (BMI≥25) as an outcome.” |
|  |  | (*c*) If relevant, consider translating estimates of relative risk into absolute risk for a meaningful time period | - |
| Other analyses | 17 | Report other analyses done—eg analyses of subgroups and interactions, and sensitivity analyses | Table 1 and 2 |
| Discussion | | |  |
| Key results | 18 | Summarise key results with reference to study objectives | Discussion paragraph 1 “Using longitudinal data from three British birth cohorts who experienced the obesity epidemic at increasingly younger ages in adulthood, we identified large and persisting socioeconomic inequalities…” |
| Limitations | 19 | Discuss limitations of the study, taking into account sources of potential bias or imprecision. Discuss both direction and magnitude of any potential bias | Discussion, Strengths and limitations sub-section. |
| Interpretation | 20 | Give a cautious overall interpretation of results considering objectives, limitations, multiplicity of analyses, results from similar studies, and other relevant evidence | Discussion, Implications sub-section. |
| Generalisability | 21 | Discuss the generalisability (external validity) of the study results | Discussion, Strengths and limitations sub-section. |
| Other information | | |  |
| Funding | 22 | Give the source of funding and the role of the funders for the present study and, if applicable, for the original study on which the present article is based | Funding sub-section “This project is part of a collaborative research programme…” |

*Give information separately for exposed and unexposed groups.

**Note:** An Explanation and Elaboration article discusses each checklist item and gives methodological background and published examples of transparent reporting. The STROBE checklist is best used in conjunction with this article (freely available on the Web sites of PLoS Medicine at http://www.plosmedicine.org/, Annals of Internal Medicine at http://www.annals.org/, and Epidemiology at http://www.epidem.com/). Information on the STROBE Initiative is available at http://www.strobe-statement.org.
