# Supplementary material for: Socioeconomic Inequalities in Body Mass Index across Adulthood: Coordinated Analyses of Individual Participant Data from Three British Birth Cohort Studies Initiated in 1946, 1958 and 1970
Source: PLoS Med. 2017 Jan 10;14(1):e1002214. doi: 10.1371/journal.pmed.1002214 (PMC5224787; doi:10.1371/journal.pmed.1002214)
Supplement: S1 Text — (DOC) [file pmed.1002214.s012.doc]

**Text S1 Missing data appendix**

First, we compared the extent of missing SEP data in each cohort. These analyses showed that there was more missing childhood and adult SEP data in the later born cohorts (ie, in the 1970 compared with 1946 cohort); Table 1. This difference was particularly pronounced for childhood SEP (Table 1). Since missing childhood SEP data were particularly pronounced among the 1958 NCDS and 1970 BCS, we conducted additional sensitivity analyses in which missing childhood SEP data on childhood social class in the were further minimised, by incorporating additional childhood social class data which used alternative occupational classification schema (1970 classification schema for 1958 NCDS and the 1980 schema for 1970 BCS); as such, missing data was minimized at the expense of comparability of childhood social class. We then repeated our examination of associations between childhood SEP and BMI associations in each cohort. Conclusions from these and the main analyses conducted were similar (shown in Table 2).

Second, we also analysed the characteristics of those with missing BMI data. Since both the number and age ranges of BMI measurements differed in each cohort, we limited analyses to age 42/43 to aid comparison of cohort differences in missing BMI data. These analyses showed that there was more missing BMI data in the later born cohorts and that, in each cohort, there was more missing BMI data among those with lower childhood SEP (Table 1). However, there was little evidence that the association between childhood SEP and missing BMI outcome data at 42/43y differed systematically by cohort (P=0.27 for childhood SEP x cohort interaction). Higher BMI at the preceding age (ages 33-36 depending on the cohort) was also associated with greater odds of missing subsequent BMI outcome data, but there was little evidence that associations differed by cohort (P=0.6 for BMI x cohort interaction).

Table 1. Prevalence and predictors of missing socioeconomic position and body mass index data

|  | Missing data in: | |  |  |  |
| --- | --- | --- | --- | --- | --- |
|  | Childhood SEP | | Adult SEP | BMI at 42/43y |  |
| 1946 NSHD (N=5362) | 26.6% | | 44.3% | 40.0% |  |
| 1958 NCDS (N=16383) | 38.6% | | 47.2% | 39.3% |  |
| 1970 BCS70 (N=16172) | 31.9% | | 53.7% | 51.1% |  |
|  |  | |  |  |  |
| Logistic regression estimates | OR (95% CI) | | OR (95% CI) | OR (95% CI) |  |
| Sex (female ref) | 1.06 (1.01, 1.12) | | 0.7 (0.64, 0.76) | 1 (0.91, 1.09) |  |
| Birth cohort |  | |  |  |  |
| 1946 (ref) |  | |  |  |  |
| 1958 NCDS | 1.83 (1.7, 1.98) | | 1.72 (1.5) | 1.38 (1.18, 1.61) |  |
| 1970 BCS70 | 1.37 (1.26, 1.48) | | 2.13 (1.86, 2.44) | 2.67 (2.29, 3.11) |  |
|  |  | |  |  |  |
| Father’s occupational class (10/11y) |  | |  |  |  |
| Class I (ref) |  | |  |  |  |
| II |  | | 1.19 (0.99, 1.44) | 1.06 (0.86, 1.3) |  |
| III NM |  | | 1.16 (0.94, 1.43) | 1.01 (0.81, 1.27) |  |
| III M |  | | 1.38 (1.15, 1.66) | 1.25 (1.03, 1.52) |  |
| IV |  | | 1.44 (1.18, 1.78) | 1.31 (1.05, 1.64) |  |
| V |  | | 1.92 (1.53, 2.41) | 1.52 (1.18, 1.95) |  |
|  |  | |  |  |  |
| BMI at age preceding 42 (33, 34, or 36y) |  | | 1.01 (1, 1.02) | 1.02 (1.01, 1.03) |  |
|  |  |  | |  |  |

Table 2. Father’s occupational class (10/11y) and BMI across adulthood (≥20 years) in the 1946 NSHD, 1958 NCDS, and 1970 BCS British birth cohort studies: estimates from separate multilevel models, scaled to show estimated BMI differences at 26 years

|  |  | Cohort |  |  |
| --- | --- | --- | --- | --- |
|  | **Men** | 1946 NSHD | 1958 NCDS | 1970 BCS |
| N participants, observations |  | 1,901, 7,921 | 5,794, 20,453 | 4,882, 12,882 |
| Main effect: age |  | .14*** (0.01) | .22*** (0.0095) | .3*** (0.017) |
| Main effect: age^2^ |  | -.00042** (0.00014) | -.0025*** (0.00016) | -.0086*** (0.00049) |
| Main effect: SEP | Class I (ref) |  |  |  |
|  | II | .65* (0.26) | .53** (0.2) | .57* (0.25) |
|  | III NM | 0.36 (0.28) | .59** (0.22) | 0.25 (0.29) |
|  | III M | 1*** (0.25) | 1*** (0.19) | .82*** (0.24) |
|  | IV | 1.2*** (0.27) | .95*** (0.21) | .73** (0.28) |
|  | V | 1.3*** (0.33) | 1.1*** (0.23) | 1** (0.32) |
|  |  |  |  |  |
| SEP*age interactions | II | -0.0088 (0.011) | 0.017 (0.01) | -0.0047 (0.016) |
|  | III NM | -0.0014 (0.011) | .023* (0.011) | 0.021 (0.018) |
|  | III M | 0.012 (0.01) | .02* (0.0094) | .033* (0.016) |
|  | IV | 0.011 (0.011) | .028** (0.01) | .044* (0.018) |
|  | V | -0.015 (0.014) | .025* (0.011) | 0.022 (0.021) |
|  |  |  |  |  |
| Constant |  | 23*** (0.23) | 23*** (0.18) | 24*** (0.23) |
| Random effects | sd(xage) | .097*** (0.0027) | .11*** (0.002) | .16*** (0.0036) |
|  | sd(_cons) | 2.5*** (0.058) | 2.7*** (0.032) | 3.4*** (0.043) |
|  | sd(Residual) | 1.1*** (0.023) | 1.6*** (0.011) | 1.3*** (0.014) |
|  |  |  |  |  |

|  |  | Cohort |  |  |
| --- | --- | --- | --- | --- |
|  | **Women** | 1946 NSHD | 1958 NCDS | 1970 BCS |
| N participants, observations |  | 1,782, 7,830 | 5,638, 20,490 | 5,054, 15,057 |
| Main effect: age |  | .11*** (0.013) | .2*** (0.012) | .26*** (0.018) |
| Main effect: age^2^ |  | .00084*** (0.00017) | -.0018*** (0.00019) | -.0057*** (0.0005) |
| Main effect: SEP | Class I (ref) |  |  |  |
|  | II | 0.2 (0.28) | 0.33 (0.25) | .63* (0.29) |
|  | III NM | 0.024 (0.27) | 0.49 (0.27) | 0.55 (0.33) |
|  | III M | .67* (0.27) | 1.2*** (0.24) | 1.2*** (0.28) |
|  | IV | 1.2*** (0.29) | 1.2*** (0.26) | 1.4*** (0.32) |
|  | V | 1.5*** (0.43) | .93*** (0.28) | 1.9*** (0.37) |
|  |  |  |  |  |
| SEP*age interactions | II | 0.0063 (0.014) | 0.0014 (0.013) | 0.0012 (0.018) |
|  | III NM | 0.011 (0.014) | 0.018 (0.014) | -0.0063 (0.02) |
|  | III M | .045** (0.014) | .032** (0.012) | .038* (0.017) |
|  | IV | .053*** (0.015) | .04** (0.013) | 0.025 (0.02) |
|  | V | .05* (0.021) | 0.014 (0.014) | .061** (0.023) |
|  |  |  |  |  |
| Constant |  | 22*** (0.23) | 22*** (0.23) | 22*** (0.27) |
| Random effects | sd(xage) | .14*** (0.0049) | .13*** (0.0024) | .18*** (0.0039) |
|  | sd(_cons) | 3*** (0.094) | 3.2*** (0.039) | 4*** (0.047) |
|  | sd(Residual) | 1.5*** (0.03) | 1.9*** (0.013) | 1.6*** (0.015) |

Estimates and standard errors shown in parentheses; *p<0.05,**p<0.01,***p<0.001.
